# Expression of the human usherin c.2299delG mutation leads to early-onset auditory loss and stereocilia disorganization

**DOI:** 10.1038/s42003-023-05296-x

**Published:** 2023-09-12

**Authors:** Ryan Crane, Lars Tebbe, Maggie L. Mwoyosvi, Muayyad R. Al-Ubaidi, Muna I. Naash

**Affiliations:** 1 Department of Biomedical Engineering, University of Houston, Houston, TX 77204 USA; 2 Present Address: Department of Microbiology & Immunology, University of Oklahoma Health Sciences Center, Oklahoma City, OK 73104 USA

**Keywords:** Mechanisms of disease, Neurological disorders

## Abstract

Usher syndrome (USH) is the leading cause of combined deafness and blindness, with USH2A being the most prevalent form. The mechanisms responsible for this debilitating sensory impairment remain unclear. This study focuses on characterizing the auditory phenotype in a mouse model expressing the c.2290delG mutation in usherin equivalent to human frameshift mutation c.2299delG. Previously we described how this model reproduces patient’s retinal phenotypes. Here, we present the cochlear phenotype, showing that the mutant usherin, is expressed during early postnatal stages. The c.2290delG mutation results in a truncated protein that is mislocalized within the cell body of the hair cells. The knock-in model also exhibits congenital hearing loss that remains consistent throughout the animal’s lifespan. Structurally, the stereocilia bundles, particularly in regions associated with functional hearing loss, are disorganized. Our findings shed light on the role of usherin in maintaining structural support, specifically in longer inner hair cell stereocilia, during development, which is crucial for proper bundle organization and hair cell function. Overall, we present a genetic mouse model with cochlear defects associated with the c.2290delG mutation, providing insights into the etiology of hearing loss and offering potential avenues for the development of effective therapeutic treatments for USH2A patients.

## Abstract (web summary)

A gain-offunction mouse model expressing the c.2290delG mutation in usherin results in impaired ankle link protein complexes, abnormal stereocilia organization, and ultimately development of hearing deficits

## Introduction

Usher syndrome (USH) is an autosomal recessive disorder characterized by sensorineural hearing loss, vision loss, and vestibular dysfunction. It has a global prevalence of ~3 to 6 cases per 100,000 individuals (Usher syndrome: MedlinePlus Genetics) and accounts for ~50% of all hereditary deafness-blindness cases^1^. USH is a clinically heterogeneous disease that impairs the patient’s ability to interact with the external world. USH is genetically heterogeneous with ten USH genes assigned to three clinical subtypes: six genes are associated with USH1 (USH1B-G), three with USH2 (USH2A, USH2C, and USH2D), and one with USH3 (USH3A)^2^. These clinical subtypes show similar phenotypes, but vary in age of onset, severity of vision/hearing loss, and presence of vestibular dysfunction^3–7^. Given the genetic diversity of the Usher syndrome, patients show auditory and retinal phenotypes with varying onset and severity^8^. USH1 patients display an onset of retinitis pigmentosa (RP), vestibular dysfunction, and severe-to-congenital hearing loss within the first decade of life^9^. USH2 is the most common form of USH, with mutations in usherin (USH2A) being most prevalent^1^. USH2A patients display congenital moderate-to-severe hearing loss, pre-to-post pubescent onset of RP and intact vestibular function^5,10^. Mutations in the *USH2A* gene account for more than 85% of all USH2 cases and also cause isolated RP without hearing loss^1,5,10,11^. USH3 is characterized by a post lingual onset of hearing loss and variable onset of RP^8^. Vestibular function is impaired in some cases of USH3 while it is unaffected in others. To date, there is no clear understanding of the underlying pathophysiology of USH and therefore, no cure for this disease exists.

Thus far, over 1500 different mutations were identified in the USH2A gene. The c.2299delG mutation located in exon 13 is the most common, accounting for over 45% of all USH2A cases^1,12,13^. USH2A patients display congenital hearing loss that is mild-moderate in the low frequencies and severe-profound in the high frequencies (characterized as a “sloping audiogram”), pre-to-post pubescent onset of RP, and intact vestibular function^4,7,14^. However, in a few cases it has also been associated with atypical Usher syndrome, referring to patients with known USH2 mutations but an atypical clinical phenotype^15^. Additionally, cases of mutations in *USH2* genes causing non-syndromic RP with no auditory phenotype were identified, highlighting the heterogeneous nature of USH2A mutations^16^. Given the varying phenotypes in patients of syndromic USH2A, atypical USH2A and non-syndromic RP, an impact of additional factors, such as mislocalization or accumulation of mutated usherin on the onset and severity of sensorineural hearing loss in patients with RP seems plausible^16^. *USH2A* mutations, including c.2299delG, often lead to the expression of abnormally short, mutant usherin or prevent the formation of a protein product (e.g. via nonsense-mediated decay)^17^. In fact, nasal samples from patients with the c.2299delG mutation exhibited variable *USH2A* transcript profiles including many transcripts skipping exons 13 and/or 12^17^. Bioinformatic analysis of these samples suggested that the c.2299delG change disrupted an exonic splicing enhancer and created an exonic splicing silencer within exon 13 that led to skipping of exons 12 and 13 to varying degrees across samples. It is not clear how missing or truncated usherin leads to hearing/vision impairments in patients. It is also unclear why some *USH2A* mutations result in USH2, while others cause RP without hearing loss^1,18–21^.

Usherin is located at the connecting cilium (CC) of retinal photoreceptors and at the ankle links of stereocilia in the inner ear^22–24^. It has been hypothesized that usherin associates with other USH2 proteins in a multi-protein complex to form the ankle link complex at the base of developing stereocilia^6,23,25–27^. Usherin is known to interact, via its PDZ binding domain, with adhesion G protein-coupled receptor V1 (ADGRV1, USH2C, also known as VLGR1) and whirlin (USH2D), co-localizing with them at the periciliary membrane complex of the photoreceptors^6,28^. ADGRV1 and whirlin also co-localize, and likely interact with usherin during the development of the stereocilia in the inner hair cells (IHCs)^26,29^. There is evidence that myosin7a (USH1B) facilitates the transport of the three USH2 proteins to the base of the stereocilia to form the ankle link complexes^26^. Several mouse lines with mutations in USH2 and PDZD7 genes have been generated and were found to possess inner ear phenotypes, including stereocilia disorganization or degeneration and functional hearing loss^6,23,25,26,30–32^. Among them is the Ush2a knock-out (*Ush2a*^*−/−*^) mouse, which is characterized by a late-onset photoreceptor degeneration detected at 20 months of age and a moderate but non-progressive hearing impairment^33^. Although this model has been useful, it does not closely recapitulate the visual deficits seen in patients carrying *USH2A* mutations, an expected outcome since many *USH2A* mutations generate truncated products rather than null alleles^1,34,35^. Furthermore, the *Ush2a*^*−/−*^ hearing phenotype has a later onset at P120 and is restricted to higher frequency when compared to other USH2 mouse lines which exhibit early-onset hearing loss at all frequencies tested^6,23,25,31,33^. Despite the availability of these models, it remains unclear how the ankle link complex keeps the stereocilia bundle organized during development or how each protein fits in the overall complex.

To better model the genetic change seen in patients carrying the c.2299delG mutation in usherin, we generated a knock-in (KI) mouse line with a mutation equivalent to the human change (c.2290delG in mouse usherin)^13^ to allow full assessments of the retinal and cochlear phenotypes. Since the guanine deletion at position c.2299 in patients results in a frameshift followed by the addition of 20 amino acids and a premature stop codon^16^, we designed our KI model to mimic the human genetic modification, including the 20 amino acids following the mutation. We used this model to study the resulting auditory phenotype and to understand the role of usherin in the formation and function of the ankle link complex. We investigated the retinal phenotype of this model previously^36^ and found that this model exhibits vision loss associated with retinal degeneration and morphologic changes to the photoreceptor cilium; consistent with the late-onset retinitis pigmentosa phenotype observed in USH2A patients^1,13^. Biochemical studies revealed the presence of a ~110 kDa glycosylated mutant protein that was abnormally intracellularly localized in the photoreceptor inner segment and led to mislocalized ADGRV1 and whirlin. Here, we report early onset hearing loss specific to lower frequencies, persisting throughout the life of the animal. This hearing loss was associated with disorganization of stereocilia bundles in the apical portion of the cochlea. Like the retina, we also found the mutant protein trapped within the hair cell body indicative of failed, or improper, trafficking to the stereocilia during development. The current findings advance the general understanding of the role of usherin during stereocilia development and within USH2A pathogenesis; having the potential to impact USH2 diagnosis, prognosis, and treatments.

## Results

### *Ush2a*^*delG/delG*^ mice express a developmentally regulated truncated protein

The c.2299delG deletion in humans leads to a frameshift, changing a glutamine to a serine at position 767, the addition of 20 amino acids and a premature stop codon. We generated a KI mouse model (c.2290delG) for this mutation to mimic the human disease allele in the C57BL/6J background. The KI strategy included the frameshift that changed a glutamine to a serine followed by the 20 amino acid human extension, a triple (3X) FLAG tag, followed by a stop codon^36^. We also included an internal ribosomal entry site (IRES), after the stop codon followed by GFP to allow for translation of usherin and GFP under the control of the endogenous usherin promoter. USH2A is an autosomal recessive disease, therefore homozygous animals, herein referred to as *Ush2a*^*delG/delG*^, are the relevant genetic model for cochlea defects. Consistent with this, we found that cochlear phenotype in the heterozygous mice were comparable to wild type (WT), accordingly we largely present results from WT and *Ush2a*^*delG/delG*^.

**Fig. 1:**
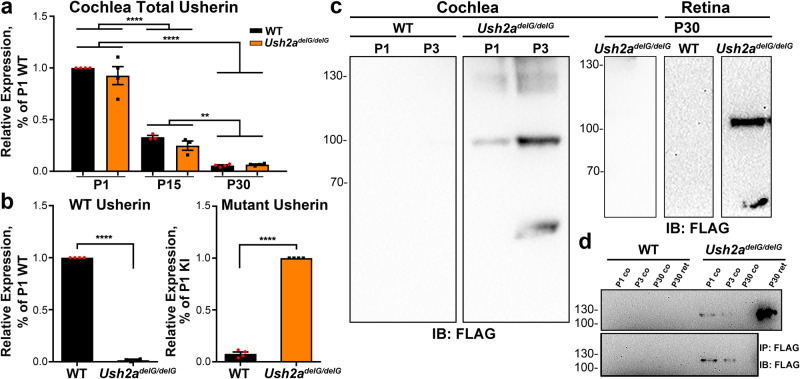
Targeted *Ush2a*^*delG/delG*^ KI results in properly regulated message and production of truncated protein in the KI cochlea. **a** Quantitative RT-PCR of total RNA isolated from the cochlea shows developmental steady state levels of usherin transcript are comparable in WT and KI at P1, 15 and 30. Values are plotted as mean ± SEM from three independent samples. Comparison between timepoints **P = 0.0047 (WT P15 vs. P30) and ****P < 0.0001 (P1 vs. P15, P1 vs. P30) by two-way ANOVA followed by Tukey post-hoc test, no significant difference found between different genotypes at the same timepoint. **b** Quantitative RT-PCR showing that WT transcript is exclusively expressed in the P1 WT cochlea (WT Usherin) while mutant transcript is only expressed in the P1 KI cochlea (Mutant Usherin). Samples were normalized to the expression levels of HPRT and then normalized to WT (in the WT Usherin panel) or *Ush2a*^*delG/delG*^ (in the Mutant Usherin panel). ****P < 0.0001 by unpaired *t*-test analysis. **c** Immunoblots of cochlea and retinal extracts from the indicated genotypes and ages were probed with anti-FLAG antibody. Mutant usherin is detected at P1 and P3 but not in P30 *Ush2a*^*delG/delG*^ cochlea. The size of mutant protein in the cochlea is comparable to its size in the retina (~110 kDa, blot on the right). No mutant protein was detected in WT cochlea or retina at all ages. **d** Immunoprecipitation from cochlea lysates further validates the presence of the mutant usherin in the *Ush2a*^*delG/delG*^ cochlea at P1 and P3. At P30 the mutant protein was no longer present. Uncropped blots used to generate c and d are presented in Fig. S6.

USH2 proteins take part in the early development and organization of the cochlear inner hair cells (IHCs) and outer hair cells (OHCs) by forming part of the ankle link^29,37^. While the exact role of each USH2 protein has yet to be identified, the localization of these proteins to the base of the stereocilia has been well studied^23,24,29,33,38^. The ankle link proteins are present during early development but gradually disappear as the stereocilia mature, however, the exact timeline of that reduction and eventual absence of the proteins has varied between studies depending on the ages enrolled in these studies, for example refs. ^26,29^. Since usherin is understood to be part of the ankle link complex, we first determined the developmental expression pattern in WT and *Ush2a*^*delG/delG*^ cochleae at postnatal day (P) 1, 15, and 30 using qRT-PCR (Fig. 1a). Using primers that amplify both the mutant and WT *Ush2a*, we observed comparable levels of total usherin transcripts from the KI and WT cochleae at all ages studied and we found transcript levels significantly decreased from P1 to P15 and were undetectable by P30 for both KI and WT (Fig. 1a). Using primers specific to either WT (Fig. 1b, left) or mutant Ush2a (Fig. 1b, right), we confirmed that WT transcript is absent in the *Ush2a*^*delG/delG*^ and vice-versa. Samples were normalized to the expression levels of HPRT and then normalized to WT (left) or *Ush2a*^*delG/delG*^ (right). Immunoblot analysis using anti-FLAG antibody targeting the truncated usherin detected the mutant protein only in P1 and P3 KI cochlear lysates (Fig. 1c, left two blots) but absent at P30 (Fig. 1c, right blot). The KI protein in cochlear extracts ran at a molecular size of ~110 kDa, the same size detected in the P30 *Ush2a*^*delG/delG*^ retina (Fig. 1c, far right blot). A lower band was occasionally detected in the KI lysates from the cochlea and retina as shown for the P3 cochlea and P30 retina, likely a specific proteolytic product. No FLAG band was detected in P1 or P3 WT cochleae or in P30 WT retina (Fig. 1c). The absence of the mutant protein in P30 *Ush2a*^*delG/delG*^ cochlea confirms that regulation of expression of the mutant usherin protein is comparable to that of the WT protein, which is reported to disappear at P15^24^. To further investigate our findings, immunoprecipitation with an anti-FLAG antibody was performed (Fig. 1d). The mutant protein was precipitated from P1 and P3 *Ush2a*^*delG/delG*^ cochleae, as well as from P30 *Ush2a*^*delG/delG*^ retina. In line with the immunoblot in Fig. 1c, no mutant protein was precipitated from P30 *Ush2a*^*delG/delG*^ cochlea or from P1, P3 and P30 WT cochleae and P30 WT retina. These findings are consistent with the suggestion that usherin is important in the development of stereocilia bundle and is less involved with the mature hair cells.

### Expression of *Ush2a*^*delG/delG*^ leads to increased ABR thresholds in the lower frequency range

**Fig. 2:**
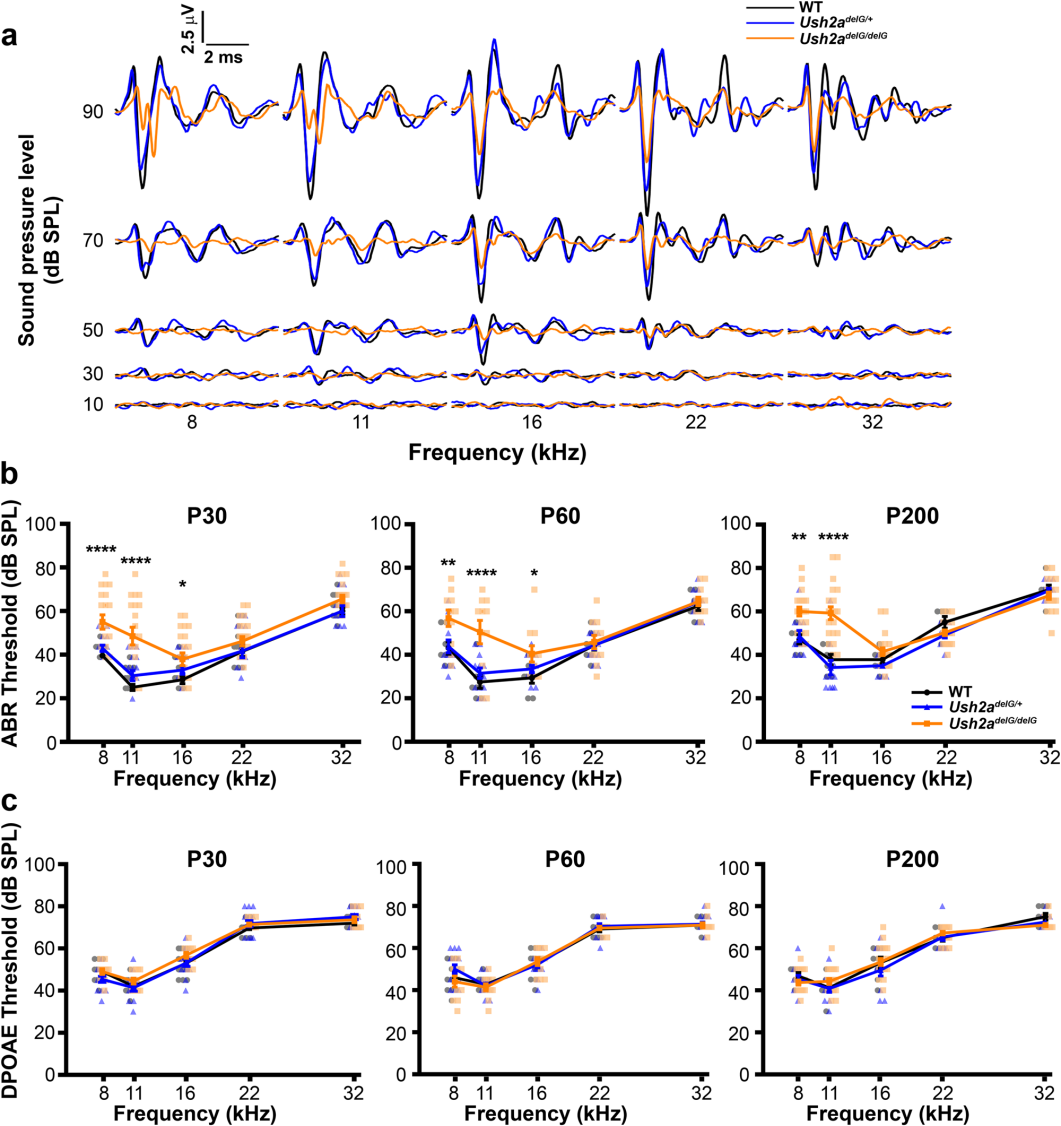
*Ush2a*^*delG/delG*^ exhibit elevated response thresholds in the lower frequency range. **a** Average ABR pure tone waveforms for WT (black), *Ush2a*^*delG/+*^ (blue) and *Ush2a*^*delG/delG*^ (orange) mice at P30. **b** ABR response threshold at different test frequencies measured in WT, *Ush2a*^*delG/+*^ and *Ush2a*^*delG/delG*^ mice at P30, P60, and P200 timepoints. Elevated threshold levels observed in *Ush2a*^*delG/delG*^ for lower frequencies (8 and 11 kHz) at all timepoints. Plotted are means ± SEM. *P = 0.0186 (P30, 16 kHz), *P = 0.0346 (P60, 16 kHz), **P = 0.0064 (P60, 8 kHz), **P = 0.0011 (P60, 8 kHz), ****P < 0.0001 by two-way ANOVA followed by Tukey post-hoc test. For all tested frequencies, P30: WT (*N* = 13), *Ush2a*^*delG/+*^ (*N* = 12), and *Ush2a*^*delG/delG*^ (*N* = 17); P60: WT (*N* = 8), *Ush2a*^*delG/+*^ (*N* = 13), and *Ush2a*^*delG/delG*^ (*N* = 11); P200: WT (*N* = 7), *Ush2a*^*delG/+*^ (*N* = 12) and *Ush2a*^*delG/delG*^ (*N* = 20). **c** DPOAE response threshold at different test frequencies measured in WT, *Ush2a*^*delG/+*^ and *Ush2a*^*delG/delG*^ mice at P30, P60 and P200 timepoints. For all tested frequencies, P30: WT (*N* = 15), *Ush2a*^*delG/+*^ (*N* = 14), and *Ush2a*^*delG/delG*^ (*N* = 11); P60: WT (*N* = 11), *Ush2a*^*delG/+*^ (*N* = 14), and *Ush2a*^*delG/delG*^ (*N* = 11); P200: WT (*N* = 8), *Ush2a*^*delG/+*^ (*N* = 12) and *Ush2a*^*delG/delG*^ (*N* = 20). Individual traces are shown in Fig. S1.

First, we wanted to assess the functional consequences of the expression of the mutant protein. Auditory brainstem response (ABR) and distortion product otoacoustic emission (DPOAE) tests were performed on WT and KI animals at P30, 60, and 200. *Ush2a*^*delG/delG*^ animals had significantly elevated ABR pure tone thresholds in comparison to their age-matched WT and *Ush2a*^*delG/+*^ littermates (Fig. 2a, b). The average tone waveforms at P30 were plotted and overlaid to best visualize the functional loss in *Ush2a*^*delG/delG*^ animals: WT (black), *Ush2a*^*delG/+*^ (blue) and *Ush2a*^*delG/delG*^ (orange) (Fig. 2a). The elevated thresholds were specifically localized to the lower frequency ranges. This significant elevation in threshold began as early as P30, the first time point tested, and persisted to P200, the last time point investigated (Fig. 2b). Hearing loss was consistent at all three time points, suggesting that the defect is developmental and not progressive.

DPOAE tests, however, showed no differences between WT and KI at any of the tested timepoints (Fig. 2c). Unchanged DPOAE thresholds indicate that the functional loss detected by ABR is not caused by OHC structural changes but rather is primarily due to IHC changes.

There was some variability in the threshold measurements for the ABR pure tone recordings within *Ush2a*^*delG/+*^ and *Ush2a*^*delG/delG*^ mice in comparison to WT (Fig. S1a). More individual mouse threshold measurements (Fig. S1a, gray lines) deviated from the average (Fig. S1a, black line) measurement for the *Ush2a*^*delG/+*^ and *Ush2a*^*delG/delG*^ mice in comparison to WT. This variability was greatest in the *Ush2a*^*delG/delG*^, particularly at P30, but most of the ABR threshold values were higher than their WT counterparts (see Supplementary Table 3). Unlike the ABR pure tone testing, the DPOAE tests had an overall lower threshold variability (Fig. S1b).

### Disrupted stereocilia bundle organization occurs at the apical regions of the cochlea in KI mice

**Fig. 3:**
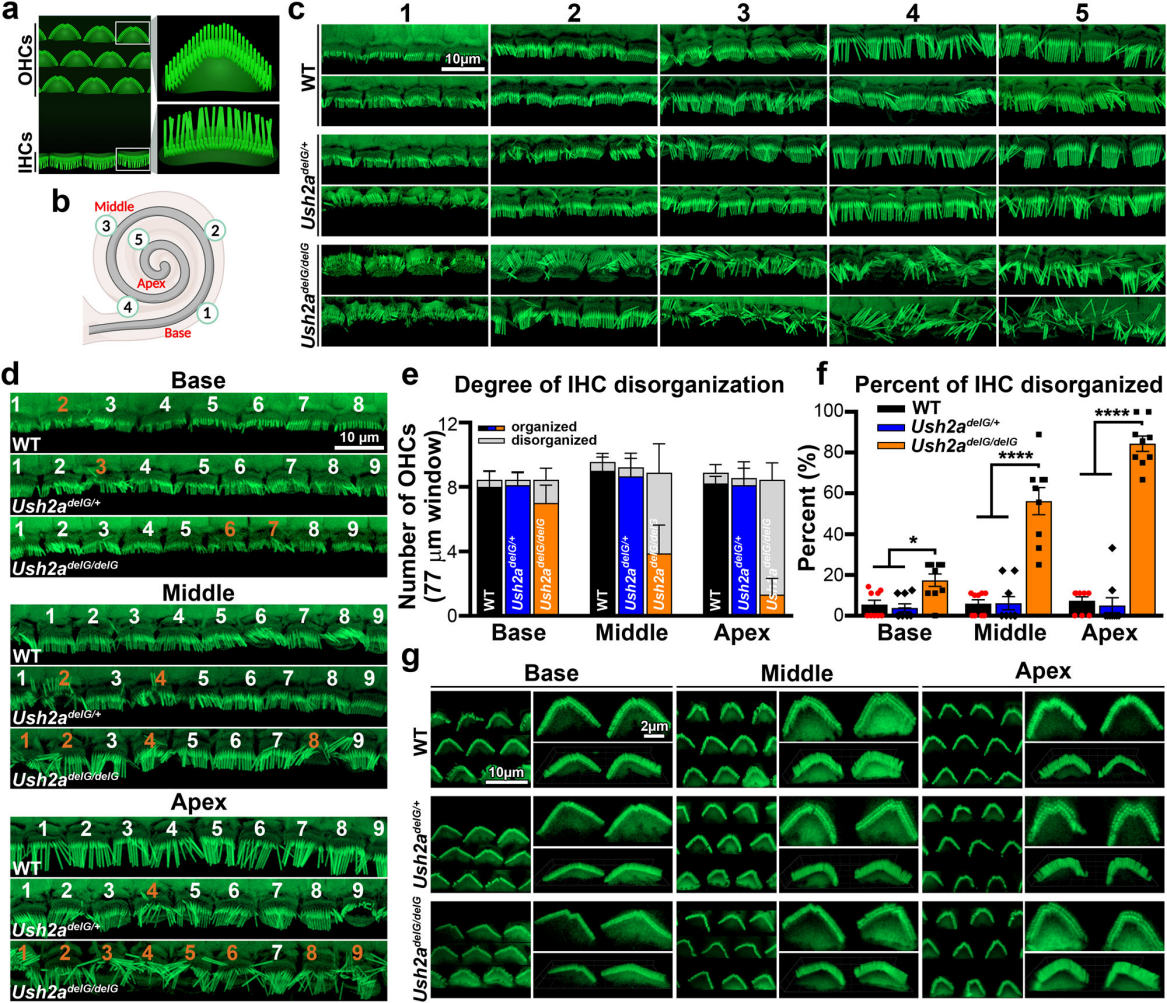
*Ush2a*^*delG/delG*^ cochlea have disorganized inner hair cell bundles at P200. **a** Schematic drawing of hair cell organization in the Organ of Corti with 1 row of IHCs and 3 rows of OHCs. **b** Schematic depicting the cochlear spiral with labeled regions from 1–5 from base to apex. **c** Staining with phalloidin (green) of the IHCs in WT, *Ush2a*^*delG/+*^ and *Ush2a*^*delG/delG*^ mice at P200 shows disrupted organization of stereocilia bundles in the apical portion of the *Ush2a*^*delG/delG*^ cochlea, notably in regions 4 and 5. **d** IHC stereocilia bundles at the basal, middle, and apical turns are numbered in white (organized) and orange (disorganized). **e** The number of disorganized versus organized bundles in a 77 µm image is plotted (mean ± SEM). **f** Shown is the total percentage of disorganized bundles per region (mean ±SEM). *P = 0.0439 (*Ush2a*^*delG/delG*^ vs. WT, Base), *P = 0.0192 (*Ush2a*^*delG/delG*^ vs. *Ush2a*^*delG/+*^), ****P < 0.0001 by two-way ANOVA followed by Tukey post-hoc test. 9 images were used for each region/genotype. **g** Phalloidin staining (green) of OHCs in P200 WT, *Ush2a*^*delG/+*^ and *Ush2a*^*delG/delG*^ whole mount cochlea shows no obvious abnormalities.

Hair cells of the cochleae are organized in continuous rows from the base to the apex with one row of IHCs and three rows of OHCs (diagramed in schematic, Fig. 3a). The stereocilia associated with these cells consist of highly organized parallel actin filaments that orient in a stair-like pattern, with three rows of increasing lengths from the center outward^39, 40^. The stereocilia rows are interconnected by a thin extracellular protein filament, known as tip link, which allows for mechanoelectrical transduction (MET) of the hair cell bundle to take place^41^. Any disruption in the organization will prevent the MET process, and thus a dampened signal (or no signal) will be transmitted to the brain when there is a sound in the environment.

After discovering the elevation in ABR pure tone thresholds at lower frequencies, we decided to determine whether there was any noticeable correlation between IHC or OHC stereocilia bundle organization and the functional changes. To do so, cochleae from P200 animals were collected, dissected into the different spiral turns of the cochlea (Fig. 3b) and stained with phalloidin, a marker for actin filaments. The cochlea spiral was imaged at five different locations, from base to apex, in at least 3 animals of the same genotype to evaluate stereocilia organization (Fig. 3b). The IHCs of the WT and *Ush2a*^*delG/+*^ showed no significant disruption in stereocilia bundle organization in any of the regions imaged (Fig. 3c). In contrast, the *Ush2a*^*delG/delG*^ exhibited clear disruption in organization of IHC bundles, particularly toward the apical end, regions 4 and 5, with some disruption noticed in region 3.

To quantify these findings, we counted the number of IHC stereocilia bundles that were disorganized per genotype in three regions (base-1, middle-3, and apex-5, Fig. 3d). Stereocilia bundles were counted as either organized (white numbers) or disorganized (orange numbers) (Fig. 3d). The total number of stereocilia bundles was similar across genotypes and regions. However, the number of disorganized bundles was significantly higher in P200 *Ush2a*^*delG/delG*^ cochlea (Fig. 3e, f). The disorganization was observed in all three regions, increasing in disorganization from base to apex (Fig. 3e). Over 80% of bundles in the apical region of the *Ush2a*^*delG/delG*^ cochleae were disorganized, with lower degrees of disorganization in the middle and basal regions, ~50% and ~15%, respectively (Fig. 3f).

This gradient of disorganization, peaking in the apical cochlear region in *Ush2a*^*delG/delG*^, is consistent with our observation that ABR pure tone thresholds were the most elevated at the lower frequencies. The sound frequencies detected by different regions of the cochlea have been well mapped^42^ with higher frequency sounds detected by the basal region of the cochlea and lower frequencies detected by the apical regions. To determine the extent of stereocilia disorder in *Ush2a*^*delG/delG*^, stereocilia length was measured across the three different cochlear regions. No differences were observed in the lengths of the stereocilia in any of the regions between WT and *Ush2a*^*delG/delG*^ (Fig. S2a, b).

Our longitudinal functional analyses indicated that auditory defects were present at early timepoints but were not progressive. These results suggested that structural defects quantified at P200 are likely occurring earlier. Evaluation of phalloidin-stained P30 *Ush2a*^*delG/delG*^ cochleae in fact showed disorganized stereocilia in a similar pattern as at P200 (Fig. S2c). These data suggest that the mutant usherin does not support proper developmental organization of IHCs leading to observed functional loss.

In contrast to the IHCs, no obvious disorganization of OHCs was observed at P200 in the three major regions of the cochlear spiral (Fig. 3g). This is in agreement with previously described functional assessment by DPOAE (Fig. 2c).

Closer examination of phalloidin-stained images from P200 *Ush2a*^*delG/delG*^ and WT showed a sporadic loss of OHCs in *Ush2a*^*delG/delG*^ at the apical region of the cochlea (red asterisk, Fig. S3a). However, the same observation was made in WT cochlea (red asterisk, Fig. S3a) regardless of localization within the cochlear spiral. To further evaluate the degree of OHCs loss in the KI, we counted the number of OHCs in images of whole mount cochleae stained with phalloidin taken at all regions from P200 *Ush2a*^*delG/delG*^, *Ush2a*^*delG/+*^ and WT (Fig. S3b) and no differences in total OHC count were detected between the genotypes. We next evaluated the percent of OHC loss within regions of each genotype and results showed that the percent loss is highest at the apical region regardless of genotype (Fig. S3c); though no differences in the percent of OHC loss were seen between the genotypes.

### USH2 protein localization is altered in the c.2290delG mutant

**Fig. 4:**
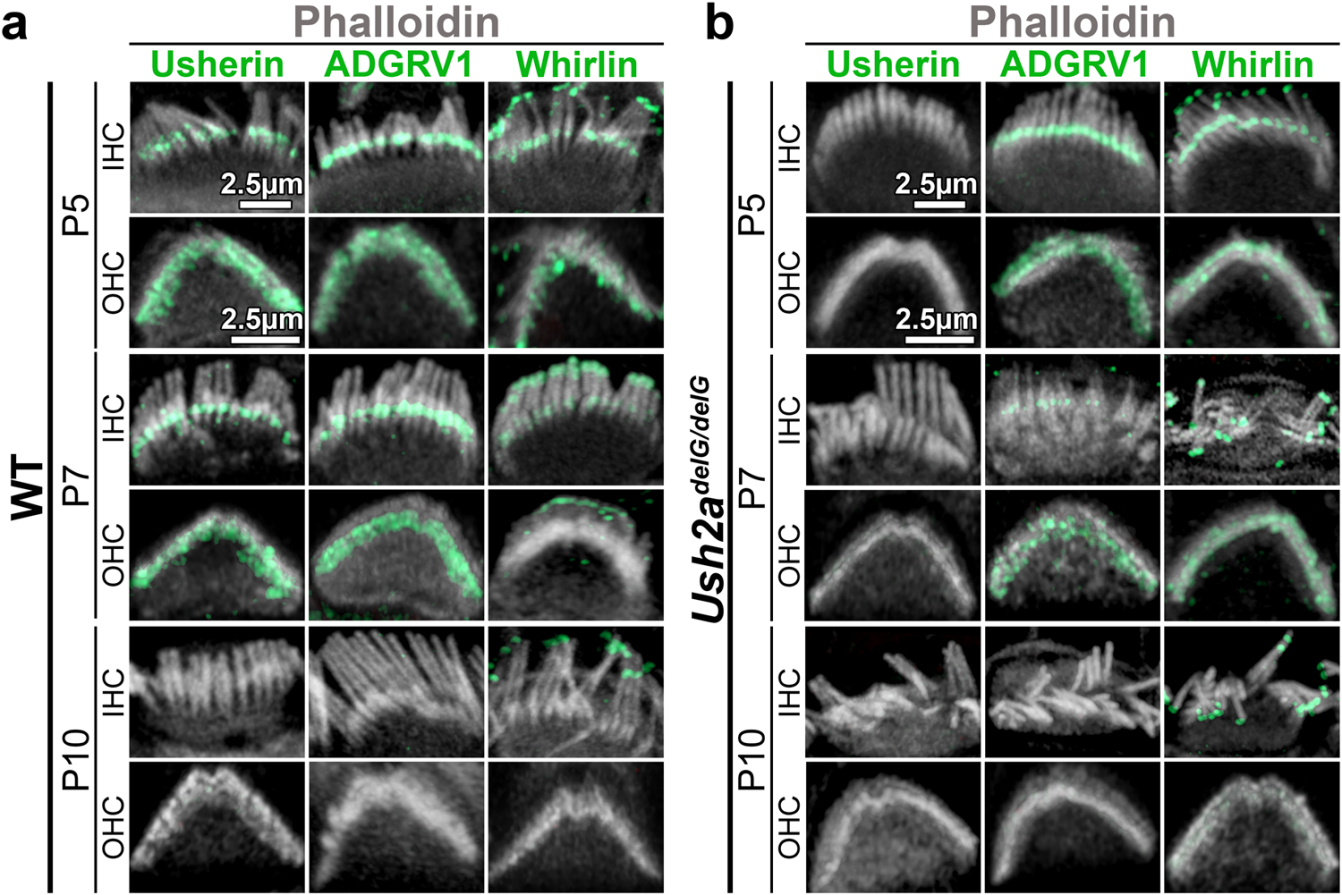
Localization of USH2 proteins during development of IHC and OHCs in WT and *Ush2a*^*delG/delG*^. Whole mount staining from the middle turn with phalloidin (gray) and usherin (green), ADGRV1 (green) and whirlin (green) at P5, P7, and P10 time points in both IHCs and OHCs for WT (**a**) and *Ush2a*^*delG/delG*^ (**b**).

During stereocilia development, the USH2 proteins usherin, whirlin, and ADGRV1 form a complex at the ankle link which provides structural support to the developing stereocilia. ADGRV1 and whirlin have been shown to localize to the base of stereocilia during early development (at the ankle link) and loss of these proteins results in stereocilia disorganization and hearing loss^23,25,26,29,31,43^. As the stereocilia mature, tip links form at the tips of the stereocilia and the ankle links regress^44^. We find this established pattern of USH2 protein localization in the WT cochlea using the stereocilia marker phalloidin (gray) and various USH2 proteins (green, Fig. 4a). At P5 and P7 WT cochleae; usherin, ADGRV1, and whirlin are found at the base of stereocilia in both IHCs and OHCs, and whirlin additionally present at the stereocilia tips (Figs. 4a and S4a, b). Consistent with the regression of the ankle links, by P10, no usherin, ADGRV1 or whirlin are seen at the base of the stereocilia (Fig. 4a and S4c), but whirlin remains properly localized at the tips of both IHCs and OHCs^26,29,43^. At P5 in *Ush2a*^*delG/delG*^ cochlea, while usherin is absent, both ADGRV1 and whirlin have similar localization patterns as in WT cochlea (Figs. 4b and S4a); indicating that trafficking of the other two USH2 proteins was unaffected by the absence of the native usherin protein. This observation of proper localization of ADGRV1 and whirlin in *Ush2a*^*delG/delG*^ cochlea is in contrast to our recent observation in the *Ush2a*^*delG/delG*^ retina, where both of these USH2 proteins were found mislocalized to the photoreceptor cell inner segment of *Ush2a*^*delG/delG*^ ^36^. However, at P7 in the *Ush2a*^*delG/delG*^ cochlea, ADGRV1 was prematurely depleted from the base of the stereocilia in both IHCs and OHCs when compared to P7 WT cochlea (Figs. 4b and S4b). This premature depletion of ADGRV1 was more prominent in IHCs where ADGRV1 was almost completely absent in the P7 *Ush2a*^*delG/delG*^ cochlea (Fig. 4b and S4b). At P10, ADGRV1 was absent in IHCs and OHCs of both WT and *Ush2a*^*delG/delG*^ mice. The stereocilia in *Ush2a*^*delG/delG*^ cochlea showed severe disorganization at this timepoint, while the stereocilia in WT cochlea were properly organized. These results suggest that usherin is not required for trafficking and final localization of ADGRV1 or whirlin to the stereocilia base of IHCs, however, as a result of its truncation, the entire ankle link complex recedes earlier than usual. This earlier loss of the USH2 ankle link complex likely contributes to the improper organization of IHC stereocilia bundles.

**Fig. 5:**
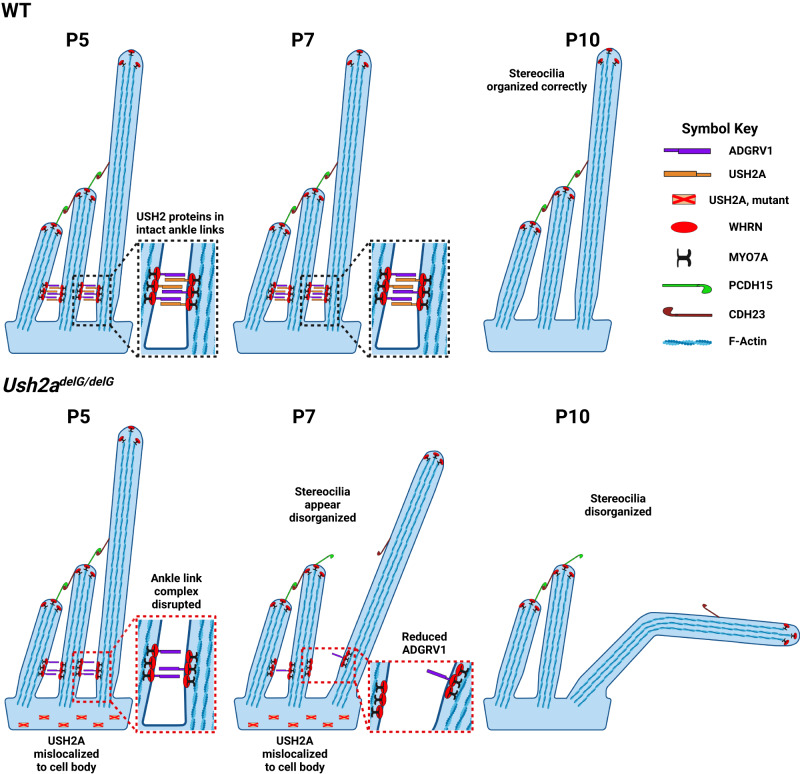
Schematic diagram summarizing the impact of mutant usherin on the structural development of hair cell stereocilia. In the WT scenario (upper panel) the ankle link complex including the USH2 proteins usherin, ADGRV1 and WHRN are present during P5 and P7 as the hair cells mature. At P10 the structural development of the hair cell stereocilia is concluded and thus the ankle link complex is no longer present. WHRN is also found at the tip of the stereo cilia at each timepoint. In the mutant scenario (lower panel) truncated usherin fails to localize to the ankle link complex. Instead, it is mislocalized in the cell body of the hair cells. This results in an incomplete formation of the ankle link complex at P5 including only two of the three USH2 proteins, ADGRV1 and WHRN. At P7, when the first structural defects start to manifest, ADGRV1 is prematurely depleted from the ankle links. At P10, the ankle link complex as well as the mislocalized truncated mutant usherin are no longer present in the hair cells. At this timepoint, the hair cell structure further deteriorated. The localization of WHRN at the tip of stereocilia was unaffected at each of the timepoints.

Since mutant usherin was absent from the connecting cilium of retinal photoreceptor and instead localized to the inner segment^36^, we checked whether the mutant protein localized outside of the expected stereocilia region. Cross sections were thus co-labeled for USH2A mutant protein (anti-FLAG labeling, red) and myosin7a (gray; hair cells outlined in pink), a marker for the hair cell body (Figs. 5 and S5). At P3 and P5 in *Ush2a*^*delG/delG*^ mice, mutant usherin is present but is abnormally distributed throughout the cell body and is absent from the base of stereocilia (Figs. 5 and S5). By P7, mutant USH2A protein is reduced in IHCs (Fig. S5), and by P10, it is no longer detected in either IHCs or OHCs (Figs. 5 and S5). As expected, GFP exhibited a similar temporal expression pattern to mutant USH2A; as expected since both are regulated by the same endogenous *Ush2a* promoter. Collectively, these results show that the mutant protein is expressed but, unlike WT usherin, lost its native localization at the base of the stereocilia.

## Discussion

The current report describes the effects of the usherin c.2290delG mutation on the function and organization of cochlear stereocilia. The results described complement the recently reported visual phenotype of this model^36^. We found that the expression of c.2290delG mutant usherin results in congenital hearing defects consistent with the known role of usherin in the formation of developmentally essential ankle links in hair cells.

The c.2299delG mutation in patients leads to heterogeneous phenotypes ranging from mild to moderate hearing loss in the low frequencies and severe to profound hearing loss in the higher frequencies; which is characterized as a “sloping audiogram” and RP^14,45^. In some patients, it has also been associated with atypical Usher syndrome with patients displaying an atypical USH2 phenotype, as well as with non-syndromic RP^16,46^. Previous studies investigating a vestibular phenotype in USH2 patients only found a mild loss of vestibular response, with no overall effect on balance^14,47^. However, a more recent in-depth study regarding the vestibular phenotype found that some of these USH2 patients (~17%) had saccular dysfunction caused by cervical vestibular evoked myogenic potentials, indicating that perhaps the vestibular phenotyping data thus far has not fully flushed out the true phenotype in patients^48^. The vestibular phenotype for this model has not been investigated in this report. In most USH models, a vestibular phenotype can be deducted from circling behavior. The knock-in mice did not display this circling behavior, indicating that there is no detectable vestibular phenotype in our model. Hearing loss is also heterogeneous among family members carrying the c.2299delG mutation in usherin. Some cases presented with moderate to severe hearing loss associated with RP while others had mild and progressive hearing loss associated with RP^49^. In the case of patients carrying two different mutations in *USH2A*, defined as heterozygous compound patients, there is a large range of symptoms displayed in hearing loss alone with some patients exhibiting slight, moderate, or severe sensorineural hearing loss; some cases being progressive while others are persistent^50^. It has been shown that *USH2A* homozygous patients expressing a truncating mutation, such as the c.2299delG, develop significantly more severe hearing impairments throughout life compared to those expressing compound heterozygous mutations where one allele has a truncating mutation while the other allele has a non-truncating mutation, or patients with two non-truncating mutations^51^.

The model described in this report addresses the specific effect of the c.2290delG mutation on cochlear development and function in a controlled genetic background and in the absence of other variables that could influence the disease phenotype. It is not surprising that the observed cochlear phenotype in the *Ush2a*^*delG/delG*^ model differs from that observed in patients, given the high variabilities in genotype-phenotype correlation observed in USH2A patients carrying the c.2299deG mutation in usherin and the differences between the mouse and humans. Many studies reported a high level of phenotypic variabilities in these patients due to many reasons. First, the large size of the *USH2A* gene and product present a challenge in identifying other variants of uncertain significance apart from the disease-causing mutations. Usherin spans 800,503 nucleotides and contains 73 exons (NM_206,933.2), including the cochlear-specific exon 71^24,52^. The presence of these other variants can adversely influence the phenotype-associated with a particular disease mutation. This makes genetic diagnosis of patients difficult and costly. Second, compound variables within a patient’s diagnosis; attribution of a specific phenotype to a specific mutation due to the presence of biallelic variants in *USH2A* and that a specific mutation can cause either syndromic USH2 or non-syndromic RP^34,53^. Syndromic cases are homozygous mutations while non-syndromic cases are compound heterozygotes with either hearing impairment or loss of vision^54^. However, while compound heterozygous mutations associated with non-syndromic hearing impairment or vision loss are better described, it is still not exactly clear whether all compounded heterozygous mutations can also result in syndromic USH2^55^. Next, is the differences due to the comparison between mouse and human, in which those differences could be due to a varying set of USH2A isoforms present in mouse and human. In human, a short (isoform A) and a long (isoform B) isoform are described^52,56^. Isoform B was found to be the one that associates with ADGRV1 and whirlin; leaving the question of other potential shorter isoforms, their function and subcellular localization open. Added to the early termination caused by the mutation, aberrant differential splicing may also be taking place within mouse and human^17^. In that case it may help explain why the protein acts differently and thus why we observe a different phenotype than typical USH2A in patients. Although unlikely, another aspect that may play a role in the phenotypic variation from that seen in humans is the presence of a 3X FLAG tag on the KI mutant protein that could impact its role in the developing stereocilia.

The controlled genetic background in our mouse model excludes the influence of the high variability seen in patients, and thus allowed us to make phenotypic observations that can be traced back specifically to the c.2299delG mutation and resulting expression/mislocalization of the truncated usherin. The studies using this mouse model demonstrated the precise impact of the c.2290delG mutation on the ankle links of IHCs, causing premature depletion of ADGRV1 and subsequent premature disassembly of this key complex. We pin-pointed the most devastating effect on stereocilia function is at the apical region of the cochlea, resulting in a significant, low-frequency specific hearing impairment. Thus, the *Ush2a*^*delG/delG*^ model confirms the role of usherin during cochlear development, how the c.2290delG mutation interferers with this role, and which areas of the cochlea show the strongest structural and functional impairment.

Consistent with patient’s phenotypic heterogeneity, we observe a degree of variability in ABR recordings in the KI mice. The patient’s variability has been attributed to genetic background and environmental factors^1,14,16,36^. However, we think that there is an inherent characteristic of this mutation that contributes to phenotypic variability since mutant mice are inbred and are raised under a controlled environment.

Prior to this study, the only available mouse model to study USH2A was the usherin-null (*Ush2a*^*−/−*^) mice^33^. Interestingly, the *Ush2a*^*−/−*^ mouse model displayed different auditory defects than our model. At 4–7 months of age, *Ush2a*^*−/−*^ mice were found to have elevated DPOAE thresholds at high frequencies (30-KHz) which correlated with significant OHC loss in the basal cochlea at 7 months^33^. No structural abnormalities in IHC were observed in these animals and no ABR recordings were reported, so it is not clear whether functional defects in IHCs might have been present. In contrast, in the *Ush2a*^*delG/delG*^ model, the apical IHCs were primarily affected, with structural disorganization of stereocilia bundles and elevated ABR thresholds detected at all timepoints examined. While we quantified structural and functional results at P30 and P200, the structural defects in IHC stereocilia organization are visible as early as P7 (e.g. Fig. 4b) suggesting that defects in IHCs of the c.2290delG model are developmental. A recent follow-up study in the *Ush2a*^*−/−*^ mice reported a similar finding of high-frequency hearing loss using complex behavioral acoustic processing tasks^57^. Along with high-frequency hearing loss in the homozygous KO mice, they also observed mild low-frequency hearing loss on complex tasks in the heterozygous mice. Thus, suggesting that some human carriers may also suffer from low-frequency hearing loss which has previously been undetected. While it has been widely observed that carriers of heterozygous *USH2A* mutations in mouse models are asymptomatic when it comes to hearing loss^31,32^, there has been some recent evidence that this is not always the case in regards to USH2A patients^58^. In addition to the mouse model, a zebrafish model was developed. The *ush2a*^*−/−*^ zebrafish model displayed significant non-progressive hearing loss starting at 10 days post fertilization (dpf)^59^. Zebrafish KI models, *ush2a*^*rmc1*^, *ush2a*^*b1245*^, and *ush2a*^*u507*^, all of which result in the expression of truncated mutant usherin were used in studying USH2A pathogenesis^60,61^. While the study of *ush2a*^*rmc1*^ and *ush2a*^*b1245*^ focused on retinal phenotype, a startle response test was performed at 5 dpf on *ush2a*^*u507*^ and showed no significant hearing impairment^61^.

Although not fully characterized, *Ush2a*^*delG/+*^ mice did not display any abnormalities when compared to WT mice. Though a slight elevation in the low-frequency range of <5 dB was observed at P30, a difference that was lost by P60 and P200, wherein the WT mice had equal or higher thresholds than the heterozygous mice.

Usherin and ADGRV1 bind to intracellular whirlin and PDZD7 to form complexes at the ankle link in the sensory hair cells and at the periciliary region of photoreceptors^24,26,62,63^. Absence of any USH2 proteins (ADGRV1, whirlin, or usherin) leads to hair cell bundle disruptions and severe hearing loss^26,33,64^, and in some cases defective hair cell bundle elongation and maintenance^1,24,26^. Usherin’s localization to the ankle link of hair cell stereocilia is only present during early development and expression of usherin is lost by P15^24,33^, findings supported by our studies. This transient expression of usherin is consistent with the process of stereocilia development wherein ankle links stabilize the base of the stereocilia at early ages, but as lateral connections and tip links mature, the supportive scaffolding at the ankle links disassembles^44^.

The timing of expression and localization of usherin points to a structural role during development to support the organization and formation of the stereocilia bundles. However, it is still unclear how usherin along with its interacting partners, participates in this process. In the retina of *Ush2a*^*delG/delG*^ animals, all USH2 proteins, usherin mutant protein, ADGRV1, and whirlin, were mislocalized away from the connecting cilium^36^. In the case of the cochlea, no mislocalization of these two usherin binding partners, ADGRV1 and whirlin, was observed at P5. While mutant usherin in our KI model loses its specific localization, ADGRV1 and whirlin retain their proper localization at the ankle links (and at nascent tip links for whirlin). However, at P7 ADGRV1 was found to be reduced at the ankle links, indicating a premature depletion of ankle link complex proteins in the mutant animals. These findings further highlight the interplay of the USH2 proteins in organizing the ankle link complex. Previous studies showed that both usherin and whirlin are either reduced or completely absent at the ankle links in the knock-out (KO) models of USH2^6,26,29^. Interestingly, the localization of whirlin at the tips of the stereocilia was not affected in any of those models. For ADGRV1 varying results were obtained. While it was found to be depleted from the ankle links in the whirlin KO model^6^, in one study ADGRV1 was reported to be reduced in the *Ush2a*^*−/−*^ mice at the ankle links^6^ while in another study it was shown to be mislocalized to the stereocilia tips^29^. In PDZD7 knockout mice, USH2 proteins were drastically reduced at the ankle links while their localization at the photoreceptor CC was not affected at all^23^. Results from these studies and the ones obtained in our KI model highlight the fact that USH2 complexes at the ankle link and periciliary region at the base of the photoreceptor CC, while sharing many commonalities, are organized and regulated differently. Our results suggest that truncated usherin weakens the stereocilia ankle links, leading to impaired stereocilia bundle. Although the ankle links are absent in the adult cochlea, they do play a critical role in early stabilization of the stereocilia to facilitate proper formation of the lateral connections and tip links that support proper stereocilia structure. Importantly this supports proper mechanoelectrical transmission and ultimately auditory signaling. Although direct testing of ankle link stability was not evaluated here for technical reasons, this idea has been proposed in prior studies of the *Adgrv1*^*−/−*^ animals^29^. Misalignment of stereocilia in the IHCs and the resulting increased distance between the tips of adjacent stereocilia also suggest lateral link and tip link formation (essential for mechanoelectrical transmission) may be impaired in the *Ush2a*^*delG/delG*^, likely due to the lack of properly formed ankle links during development. This provides a connection between developmental problems in IHC structure and the chronic but not progressive impairments in auditory function. All three USH2 proteins, usherin, whirlin, and ADGRV1, play a role during the development of IHCs, but our results suggest that the structural stability that usherin provides at the ankle link may be especially important for the longer stereocilia found in the apical region of the cochlea, which are responsible for low-frequency sound perception. This would also be consistent with our finding that OHCs were unaffected, as they have significantly shorter stereocilia than IHCs. Potential follow-up tests on the integrity of the stereocilia following auditory challenges in vivo and assessing the functional capabilities of the animals before and after such challenges might provide some understanding of the stability of stereocilia bundles lacking usherin.

On a molecular level, the loss of stereocilia alignment in the *Ush2a*^*delG/delG*^ cochleae are likely an outcome of the truncation that eliminates a large part of the extracellular portion of usherin, the anchoring transmembrane domain, and the cytosolic domain. These regions are not only important for stabilizing interactions with other USH proteins but also link the stereocilia to the cytoskeleton^65^. Some mechanistic insight into c.2299delG-associated disease and into differences between IHCs and OHCs can be gained by comparing outcomes in the *Ush2a*^*−/−*^ null with those from the *Ush2a*^*delG/delG*^ disease model. The truncated usherin could increase overall cellular stress (for example by inducing a cellular unfolded protein response), but this seems unlikely, since we do not observe overall IHC degeneration and more severe auditory defects. Another possibility is that the truncated, abnormally intracellularly localized usherin may destabilize the ADGVR1 and whirlin complexes at the ankle link even more than they are destabilized by the absence of usherin. In the *Ush2a*^*delG/delG*^ cochlea, ADGRV1 localizes correctly at the base of the stereocilia up until P5, however, it is depleted prematurely from the base as early as P7. The localization of whirlin was not affected at any investigated timepoint. This was different from the *Ush2a*^*−/−*^ animals, in which both ADGRV1 and whirlin were absent from the stereocilia base. Given that the truncated usherin neither includes the motifs required to interact with ADGRV1 and whirlin nor is present at the stereocilia at any of the investigated timepoints; it seems unlikely that the localization of ADGRV1 (until P5) and whirlin at the stereocilia in the *Ush2a*^*delG/delG*^ cochlea is mediated via a direct interaction with truncated usherin. It might be that the difference in the localization of ADGRV1 and whirlin observed in *Ush2a*^*delG/delG*^ mice when compared to *Ush2a*^*−/−*^ mice is one of the factors in why the *Ush2a*^*delG/delG*^ disease model differs so much from that observed in the *Ush2a*^*−/−*^ null mice. In addition, the truncated usherin is distributed throughout the cell body and absent from the stereocilia of HCs. This mislocalization to the cell body likely impairs the formation of tip links and lateral links. Future biochemical studies are needed to resolve these possibilities.

While there is no cure available for USH patients, some symptom-alleviating methods are currently used and some therapeutic strategies are in development. Patients with USH2 mutations often have mild enough hearing loss that the use of hearing aids can provide a substantive benefit although it does not alleviate the underlying problem nor provide full correction of hearing deficits. One of the most promising therapeutic strategies in development is gene therapy. A recent study was able to successfully use an AAV vector to treat genetic hearing loss associated with mutations in *TMC1*, the protein responsible for forming the mechanosensory transduction channel in both mice and humans^66^. They showed a return to WT hearing levels in all frequency ranges and an improved hair cell survival. As far as an example with closer relationship to USH2A, AAV has been evaluated in the whirler Usher syndrome model^67^. Whirlin was delivered via posterior semicircular canal injection of AAV in neonatal mice resulting in improved vestibular hair cell structure and function as well as some improvement in auditory function and inner hair cell development. While this procedure was successful in partially rescuing the structure and function, differences in the development of the cochlea in mice versus humans highlight challenges translating this approach to the clinic. The mouse inner ear maturates postnatally and does not fully start to function until around P12, providing an early postnatal window for possible therapy. Unfortunately, the human inner ear is fully mature at birth and thus effective intervention would likely need to occur prior to birth. Thus, while therapeutic effectiveness might be found in mice, it is concerning whether these approaches will be as effective in patients via postnatal treatment.

In conclusion, by using the *Ush2a*^*delG/delG*^ model, we have demonstrated that the disease in patients carrying this mutation is likely multifactorial; due to gain-of-function effects from the truncated protein and loss of partners, leading to impaired ankle link protein complexes, abnormal IHC stereocilia organization, and ultimately developmental hearing deficits. Based on this, it is clear that usherin is necessary for proper cochlea hair cell stereocilia organization and function.

## Methods

### Animal model

The *Ush2a*^*c.2290delG*^ KI mouse (*Ush2a*^*delG/delG*^) was generated by inGenious Targeting Laboratory, Inc., (Ronkonkoma, NY) and was previously described^36^. Genotyping using PCR confirmed that these mice are negative for the *rd8* mutation. All animals used were on C57BL/6J background. WT and KI animals were initially found to contain the *Cdh23*^*ahl*^ allele, determined by PCR amplification and DNA sequencing of the allele region^68^. In order to remove this mutation, animals were backcrossed for several generations to obtain WT and *Ush2a*^*delG/delG*^ littermates without the *Cdh23*^*ahl*^ allele (confirmed by sequencing) and then used in this study. Both male and female animals were used. Animals were maintained in 12L:12D cyclic light at ~30 lux. For terminal experiments, mice were euthanized by CO_2_ inhalation, consistent with the recommendations of the Panel on Euthanasia of the American Veterinary Medical Association. All handling, maintenance, and experimental use of animals followed protocols approved by the Institutional Animal Care and Use Committee at the University of Houston and were performed according to the NIH and the Association for Research in Vision and Ophthalmology (ARVO) guidelines.

### Auditory brainstem response and distortion product otoacoustic emissions

Mice were anesthetized via intramuscular injection of 85 mg/kg ketamine and 14 mg/kg xylazine (Butler Schein Animal Health, Dublin, OH). Once completely anesthetized, the mouse was placed on a warmed thermostatically controlled heating pad and electrodes were placed subcutaneously at the auricle of one ear (test) and contralateral auricle (reference) with a ground electrode at the vertex of the head. For ABR tests, sound stimuli were delivered through electrostatic speakers (RP-2, PA-5, ED-1, and EC-1, Tucker-Davis Technologies). The speaker was positioned near the animal’s test ear and the sonic stimulus was delivered through the speaker as tone pips with a rise and a fall time of 0.5 ms and a total duration of 5 ms at 8, 11, 16, 22, and 32 kHz. Tone pips were delivered from 10 to 90 dB SPL with 10 dB intervals at a rate of 35 per second. ABR signals were amplified using a biological amplifier (HS4/DB4, Tucker-Davis Technologies, Alachua, FL) and digitized at 200 kHz (RP-2, Tucker-Davis Technologies).

DPOAE was measured using two speakers (F_1_ and F_2_) and probe tip microphone (type 4182, Brüel & Kjaer, Denmark) built into the speaker/microphone combo. A probe was placed in the testing ear’s external auditory canal and placed gently near or against the tympanic membrane. Sound stimuli for producing DPOAEs were two 1 second sine-wave tones of differing frequencies (F_2_ = 1.22 × F_1_). The two tones were equal in intensity and stepped up in 10 dB increments. The ear canal sound pressure was pre-amplified and digitized. A fast Fourier transformation was computed. The sound pressures at f_1_, f_2_ and 2f_1_ − f_2_ were extracted after spectral averaging from 50 serial waveform traces.

### Antibodies

Antibodies, their sources, and dilutions are listed in Supplementary Table 1.

### Whole mount immunofluorescence

Following extraction from euthanized mice, cochleae were fixed in 4% paraformaldehyde in 1X PBS overnight at 4 °C. Cochleae were decalcified in 120 mM ethylenediaminetetraacetic acid (EDTA) for various periods of time, depending on age, and stored in 1X PBS. Cochleae were then dissected to isolate the Organ of Corti and Reissner’s membrane was removed. Tissue was then blocked [1% BSA, 20% donkey serum, 0.3% Triton X-100, in 1X PBS] for 1 h at room temperature, followed by incubation in primary antibody in [1% BSA, 0.3% Triton X-100, in 1X PBS] overnight at 4 °C. Following primary antibody, tissue was washed three times for 15 min in 1X PBS, and incubated in the appropriate secondary antibody in 1% BSA in 1X PBS for 1 h at room temperature, washed 4 times for 15 min in 1X PBS and mounted using Prolong gold (Invitrogen, Waltham, MA). Images taken using Zeiss LSM 800 (Carl Zeiss Microscopy GmbH) with 63X magnification. Although all regions of the cochlea were stained, the images were all taken from the central area of all regions presented in all figures.

### IHC and OHC stereocilia quantification

Cochleae from 3-4 animals per genotype were dissected to isolate the Organ of Corti and cut into 4 pieces. Tissue was labeled with Oregon Green^®^ 488 phalloidin [1% BSA in 1X PBS] for 1 h at room temperature. Images taken using Zeiss LSM 800 (Carl Zeiss Microscopy GmbH) with 63X magnification. Multiple images (77.18 µm wide) were captured for each region, from base to apex, for each animal. To keep the total number of images quantified consistent for each region, 2–4 images per region/tissue were used for quantifying the number of disorganized verses organized IHC bundles, with a total of 9 images per region used for each genotype. The number of absent or missing OHCs were counted in each image and plotted. The number of organized and disorganized IHC stereocilia were counted using the same images. Images used for IHC length were from the same group of images. 1–2 images per region/tissue we used with a minimum of 3 images per region used for each genotype. ImageJ software was used to measure the length of the stereocilia.

### Cryosection immunofluorescence

Cochleae were fixed in 4% paraformaldehyde in 1X PBS overnight at 4 °C. Cochleae were decalcified in 120 mM EDTA for various periods of time, depending on age, and stored in 1X PBS. Cochleae were cryoprotected in increasing percentages of sucrose, in 1X PBS, in overnight incubations; 10%, 20%, and 30%. Cochleae were placed in Scigen Tissue-Plus™ O.C.T. Compound (Fisher, Waltham, MA) and put under vacuum overnight before being sectioned (10 µm) and stained.

Cochleae sections were blocked in blocking buffer (1% BSA, 5% donkey serum, 5% goat serum, 0.1% Triton X-100, in 1X PBS) for 1 h at room temperature, incubated in primary antibody in blocking buffer overnight at 4 °C, washed three times for 15 min in 1X PBS, incubated in secondary antibody in blocking buffer (diluted 1:2) for 2 h at room temperature, washed 4 times for 15 min in 1X PBS and mounted in Prolong gold. Images taken using Zeiss LSM 800 (Carl Zeiss Microscopy GmbH) with ×63 magnification.

### RNA preparation and analysis

Cochleae from WT and *Ush2a*^*delG/delG*^ mice were collected and immediately frozen in liquid nitrogen and stored at −80 °C prior to processing. Due to the bony structure of the cochleae, frozen tissues were pulverized for easier extraction. Total cochlear RNA was isolated using TRIzol reagent (Life Technologies, Grand Island, NY, USA) and treated with RNase-free DNase I (Promega, Madison, WI) according to manufacturer’s recommendations. Superscript III reverse transcriptase (Life Technologies, Carlsbad, CA) along with oligo-dT primer was used to perform reverse transcription to obtain cDNA. Quantitative real time PCR was performed using a real-time PCR detection system (C1000 Thermal Cycler, Bio-Rad Laboratories Inc., Hercules, CA) using primers complementary to the N-terminal region of Ush2a transcripts (present in both WT and KI message), primers specific to the c.2290delG mutation and human 20 amino acid extension (KI only), and primers complementary to the C-terminal region of the Ush2a transcript (endogenous only). Primer sequences used are listed in Supplementary Table 2. Values were normalized to the housekeeping gene hypoxanthine-guanine phosphoribosyl transferase (HPRT).

### Protein analysis

Frozen cochleae were transferred to cold RIPA buffer (Abcam, Waltham, MA) containing 1X protease inhibitors (Roche Applied Sciences, Indianapolis, IN). The tissue was then sonicated with 10 short bursts and incubated for 1 h at 4 °C on a rocking platform. After 15 min centrifugation at 18000 × g at 4 °C, the protein concentration of the cochlea lysate was determined via Bradford assay (Bradford reagent, Bio-Rad Laboratories Inc.). A total of 150 µg of cochlea proteins for each age/genotype were run on a 12% acrylamide gel. Retina lysates from P30 WT and KI mice were prepared using 1X PBST (1X PBS, 1% Triton X-100) containing PIN. Retinas were sonicated with 3X 10 s bursts (20 s on ice between each burst) followed by 1 h incubation at 4 °C on rocking platform and 15 min centrifugation at 18,000 × *g* and 4 °C. Protein concentration of the lysates was determined via Bradford assay and 100 µg of retinal proteins from each genotype were run on an 8% acrylamide gel. Protein transfer was achieved via Wet transfer in a Mini Trans-Blot cell (Bio-Rad Laboratories Inc.) for 1.5 h at 100 V. The Transfer chamber was filled with transfer buffer (200 mM Glycin, 25 mM Tris-Base, 20% Methanol, 0.1% SDS) and kept cold during the duration of the transfer. The membrane was incubated over night at 4 °C with FLAG antibody (1:1,000, in 1X PBS). The next day the membrane was washed 3X in 1X PBST for 10 min, incubated for 1.5 h with the secondary antibody (see Supplementary Table 1) and imaged in a ChemiDocTM MP imager (Bio-Rad Laboratories Inc.).

### Statistics and reproducibility

Total RNA message levels measured using qRT-PCR were analyzed using a one-way ANOVA with a Tukey’s post hoc comparison. ABR and DPOAE line graphs were analyzed using two-way ANOVA with a Bonferroni’s post hoc comparison. Bar graphs showing the disorganized bundle numbers counted from the immunofluorescence images were analyzed using two-way ANOVA with a Bonferroni’s post hoc comparison. The sample size of each individual experiment is stated in the manuscript. For qualitative experiments a sample size of at least three (repeated on at least three separate occasions) was chosen based on our prior work. In qualitative experiments, the changes we describe are visually evident and were repeatable through different replicates. Sample sizes are typically larger for physiological and functional measurements (such as ABD and DPOAE) where past experience has shown there is more inter-animal variability. In past functional studies, sample sizes in the range of 5–10 animals per group have been sufficient to detect (with statistical significance) differences between means on the order of ~15–20%, so we used that as a starting target goal for each sample size. In this study, since it was difficult to predict the differences between group means and within-group variance, we opted for slightly larger sample sizes. For structural studies (e.g. IHC organization and OHC cell count) on inbred genetically modified mouse models, we have historically observed much less between-animal variation than with functional studies, and sample sizes of 3–5 have been sufficient to detect a difference (e.g. increased disorganization of IHCs), so that was what we used for this study. All observation stated in the study were reproducible.

### Study approval

All handling, maintenance, and experimental use of animals followed protocols approved by the Institutional Animal Care and Use Committees and were performed according to the NIH and the Association for Research in Vision and Ophthalmology (ARVO) guidelines.

### Reporting summary

Further information on research design is available in the Nature Portfolio Reporting Summary linked to this article.

### Supplementary information

Supplementary Information

Description of Additional Supplementary Files

Supplementary Data 1

Reporting Summary

## Data Availability

All data generated or analyzed during this study are included in this article and in its supplementary information file. The source data for all graphs is provided as a compiled Excel sheet (filename: Supplementary Data 1). Uncropped blots for Fig. 1c, d are shown in Fig. S6.
