# Supplementary material for: Expression of the human usherin c.2299delG mutation leads to early-onset auditory loss and stereocilia disorganization
Source: Commun Biol. 2023 Sep 12;6:933. doi: 10.1038/s42003-023-05296-x (PMC10497539; doi:10.1038/s42003-023-05296-x)
Supplement: Supplementary file 1 — Supplementary Information [file 42003_2023_5296_MOESM1_ESM.pdf]

Supplementary Information

Expression of the human usherin c.2299delG mutation leads to early-onset auditory loss and stereocilia disorganization

Ryan Crane[1], Lars Tebbe[1], Maggie L. Mwoyosvi[1,#], Muayyad R. Al-Ubaidi[1,*], and Muna I. Naash[1,*]

[1]Department of Biomedical Engineering, University of Houston, Houston, Texas 77204, USA

[#]Current address: Department of Microbiology & Immunology, University of Oklahoma Health Sciences Center, Oklahoma City, OK 73104, USA

Classification: Biological Sciences, Cell Biology

*To whom correspondence should be addressed at: Muna I. Naash (ORCID: 0000-0002-6534-5144), mnaash@central.uh.edu; Phone: 713-743-1651 (MIN) and/or Muayyad R. Al-Ubaidi (ORCID: 0000-0002-4914-350X), malubaid@central.uh.edu; Phone: 713-743-1648 (MRA); Department of Biomedical Engineering, University of Houston, 3517 Cullen Blvd. Room 2018, Houston, TX 77204-5060.

Table of Contents

Supplementary Table 1. List of antibodies used in the study and their sources 3

Supplementary Table 2. List of primers used in the study 3

Supplementary Table 3. ABR pure tone and DPOAE average and standard deviation 4

Fig. S1. Individual response thresholds for all genotypes and ages 5

Fig. S2. *Ush2a*$^{delG/delG}$ shows normal IHC length and disorganized IHC stereocilia bundles as early as P30 7

Fig. S3. Numbers of OHCs are unaffected in *Ush2a*$^{delG/delG}$ 8

Fig. S4. Localization of Ush2 proteins throughout the development of IHC and OHCs in WT and *Ush2a*$^{delG/delG}$ 9

Fig. S5. Localization of mutant usherin using anti FLAG antibody and anti GFP antibody during development in WT and *Ush2a*$^{delG/delG}$ through different regions. 10

Fig. S6. Uncropped blots used to assemble Fig. 1c (a and b) and Fig. 1d (c, c' and c'') 11

Supplementary Table 1. List of antibodies used in the study and their sources.

Antigen	Species	Application/Concentration	Source	RRID#
F-Actin (647 tagged Phalloidin)	NA	1:1000 (IF-wholemount)	ThermoFisher (A30107)	NA
FLAG	Rat	1:200 (IF-cryo)	Biolegend (L5)	AB_2749907
FLAG	mouse	1:30 (IP)	Proteintech (66008-4)	AB_2918475
FLAG	mouse	1:1000 (IB)	Millipore Sigma (F1804)	AB_262044
GFP (488 tag)	Rabbit	1:500 (IF-cryo)	Invitrogen (A-21311)	AB_221477
Myosin7a	Rabbit	1:250 (IF-cryo)	Abcam (ab3481)	AB_303841
Usherin C-term	Rabbit	1:5000 (IF-wholemount, IB)	Dr. Jun Yang[1]	NA
Whrn	Rabbit	1:200 (IF-wholemount)	Proteintech, (25881-1-AP)	AB_2880280
ADGRV1 (Vlgr1)	Rabbit	1:250 (IF)	Dr. Uwe Wolfrum[2]	NA

Supplementary Table 2. List of primers used in the study.

Target	Primer	Sequence
total Ush2a message	forward primer	5’-CCGAATTGACCTTAGCTGTGTGGTTG-3’
total Ush2a message	reverse primer	5’-GTCCTACTTGTATCACGCTGGAGAC-3’
c.2290delG mutant message	forward primer	5’-GGGACCGTGGATGGAGACATTAC-3’
c.2290delG mutant message	reverse primer	5’-GGTGTCACACTGAAGTCCTTTGGC-3’
WT message:	forward primer	5’-GGGACCGTGGATGGAGACATTAC-3’
WT message:	reverse primer	5’-CACTGCCCTGTCTCAGTGTCACA-3’

Supplementary Table 3. ABR pure tone and DPOAE average and standard deviation.

			Tone					DPOAE				
			8	11	16	22	32	8	11	16	22	32
WT	P30	Avg	39.6	25.0	28.5	41.5	60.0	49.0	42.0	53.0	69.7	72.0
		SD	2.5	5.0	6.3	8.5	5.8	4.3	3.7	5.6	3.0	3.2
	P60	Avg	43.1	27.5	29.4	44.4	62.5	45.9	42.7	52.7	69.1	70.9
		SD	8.0	8.0	6.8	6.2	6.0	7.1	3.6	7.1	4.3	2.2
	P200	Avg	47.1	42.1	42.1	55.0	70.0	46.9	41.3	53.1	65.0	75.0
		SD	6.1	7.4	5.8	7.4	4.1	3.9	6.3	7.6	5.0	4.5
Ush2a$^{delG/+}$	P30	Avg	44.6	33.8	35.8	41.7	60.0	45.4	41.4	53.2	71.8	75.0
		SD	6.9	11.9	9.3	10.5	8.5	5.4	6.3	6.4	5.8	4.4
	P60	Avg	43.8	34.6	33.5	44.2	64.2	50.0	42.1	52.1	70.4	71.4
		SD	11.7	13.4	6.7	6.7	4.8	6.8	3.9	6.0	3.4	4.0
	P200	Avg	48.8	34.2	35.0	49.2	69.6	45.4	40.8	49.6	65.4	72.5
		SD	8.8	11.6	3.7	4.7	4.0	6.2	6.3	9.4	5.8	3.4
Ush2a$^{delG/delG}$	P30	Avg	55.0	48.5	37.9	46.2	65.6	46.9	43.8	55.6	70.6	72.8
		SD	13.3	16.7	12.1	8.6	7.0	5.1	4.3	4.8	3.1	4.1
	P60	Avg	56.8	50.5	40.5	45.9	64.5	44.1	41.4	53.6	69.5	70.9
		SD	12.5	17.8	12.3	10.2	6.5	8.6	5.5	6.4	2.7	3.8
	P200	Avg	60.3	57.1	41.1	52.6	69.5	43.8	45.8	53.5	67.3	71.0
		SD	12.1	14.7	7.6	7.9	7.1	5.3	8.3	7.3	3.8	2.6

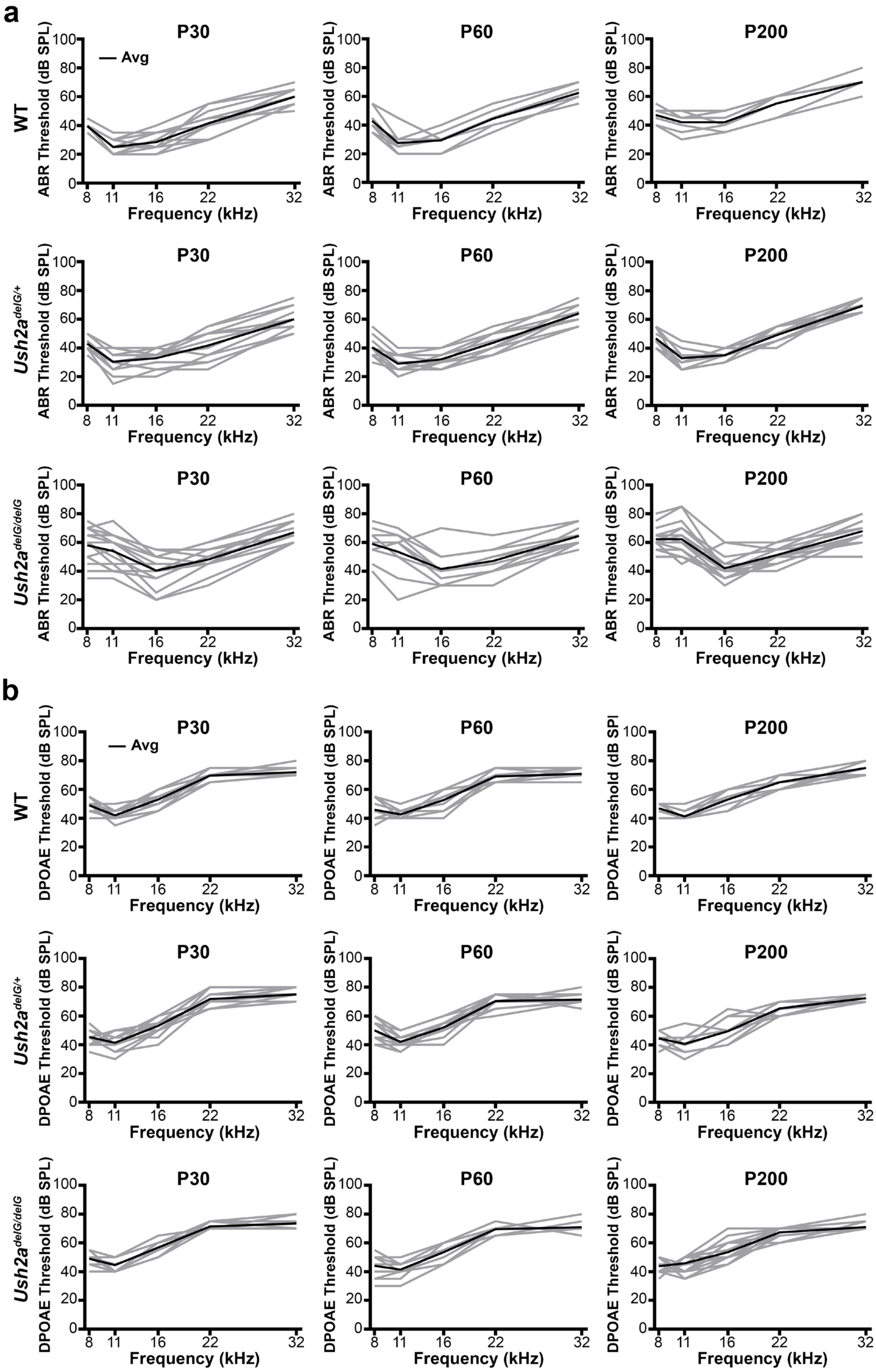
a
b
WT
Ush2a^delG/+
Ush2a^delG/delG
P30
P60
P200
Avg
ABR Threshold (dB SPL)
DPOAE Threshold (dB SPL)
Frequency (kHz)
0
20
40
60
80
100
8
11
16
22
32

Fig. S1. Individual response thresholds for all genotypes and ages. Individual response threshold at different test frequencies measured in WT, *Ush2a*$^{delG/+}$ and *Ush2a*$^{delG/delG}$ mice at P30, P60 and P200 timepoints for ABR pure tone (**a**) and DPOAE (**b**). Average measurement is in black. For ABR pure tone: WT (P30 = 13, P60 = 8, and P200 = 8); *Ush2a*$^{delG/+}$ (P30 = 12, P60 = 11, and P200 = 11); *Ush2a*$^{delG/delG}$ (P30 = 14, P60 = 10, and P200 = 17). For DPOAE: WT (P30 = 15, P60 = 11, and P200 = 8); *Ush2a*$^{delG/+}$: (P30 = 14, P60 = 14, and P200 = 12); *Ush2a*$^{delG/delG}$: (P30 = 11, P60 = 11, and P200 = 20).

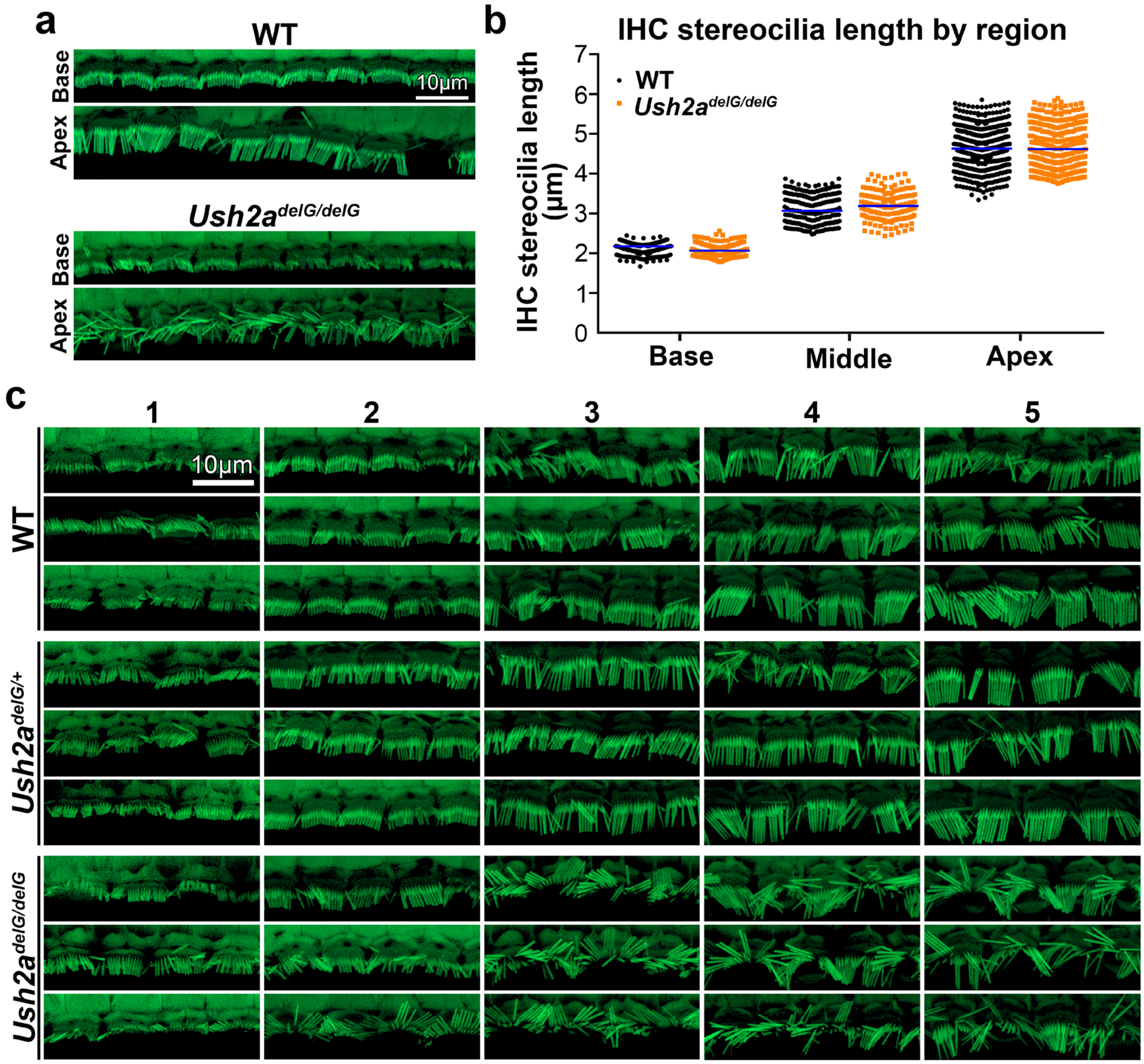

Fig. S2. ***Ush2a***$^{delG/delG}$ **shows normal IHC length and disorganized IHC stereocilia bundles as early as P30.** (**a**) Representative images of phalloidin stained IHC (green) at the indicated regions from P200 WT and *Ush2a*$^{delG/delG}$ used to measure stereocilia length. (**b**) Quantification of IHC stereocilia length from P200 WT and *Ush2a*$^{delG/delG}$ at the base, middle and apex shown to be comparable to WT stereocilia length at the three main regions. Stereocilia lengths from both genotypes increase from the base to middle and to apex. Blue line highlights mean. No statistical significance between genotypes in each region by parametric t-test. 160 – 568 stereocilia were observed per genotype coming from 3 independent cochleae. (**c**) Staining with phalloidin (green) of the IHCs in WT, *Ush2a*$^{delG/+}$ and *Ush2a*$^{delG/delG}$ mice at P30 show disrupted organization in the apical portion of the cochlea, notably in regions 4 and 5.

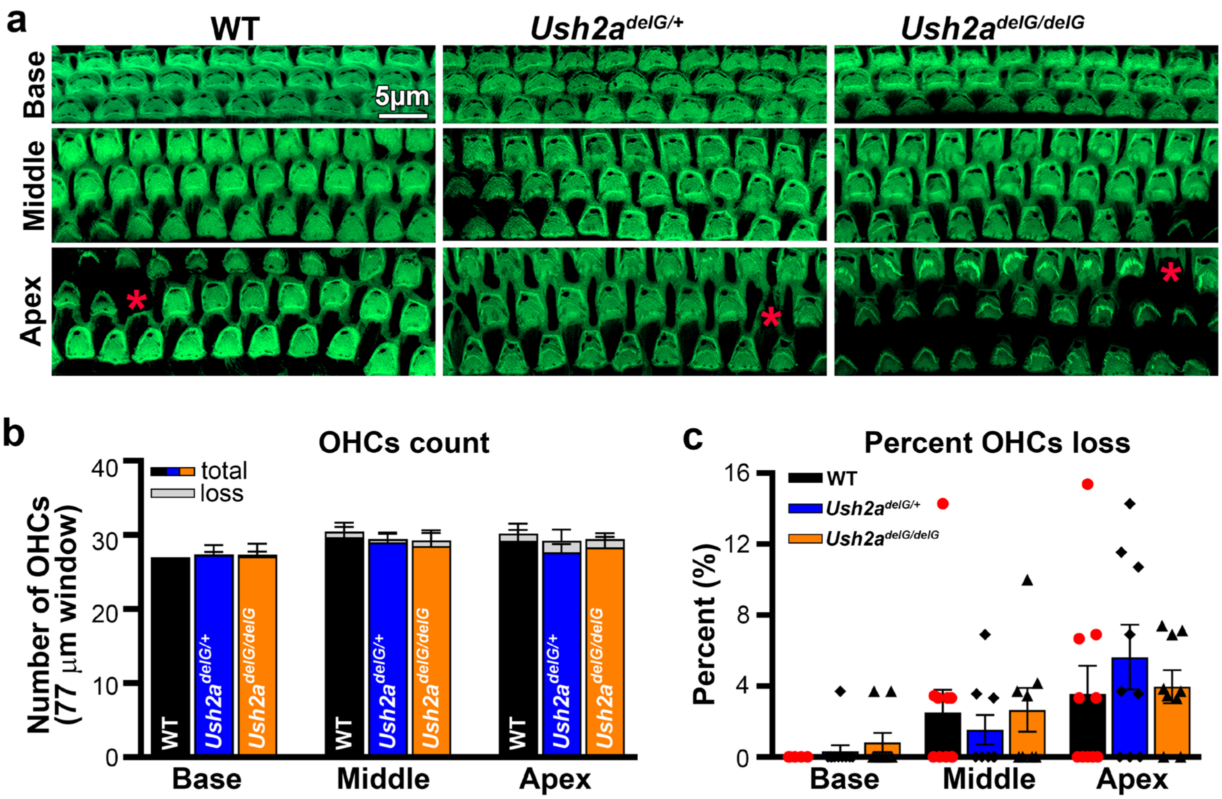

Fig. S3. Numbers of OHCs are unaffected in *Ush2a$^{delG/delG}$*. (**a**) Images of OHC of P200 WT *Ush2a$^{delG/+}$* and *Ush2a$^{delG/delG}$* cochlea stained with phalloidin (green) and highlighting absence of some OHC in all genotypes (red stars). Total OHC count appeared unaffected by the mutation. (**b**) Count of the OHCs shows no apparent differences between the genotypes, regardless of the region, with a slightly higher number in the apical region overall (mean ±SEM). No statistical significance by two-way ANOVA followed by Tukey post-hoc test was observed. Total of 9 images were used for each region/genotype. (**c**) Shown is the total percent of OHCs lost per region for each genotype.

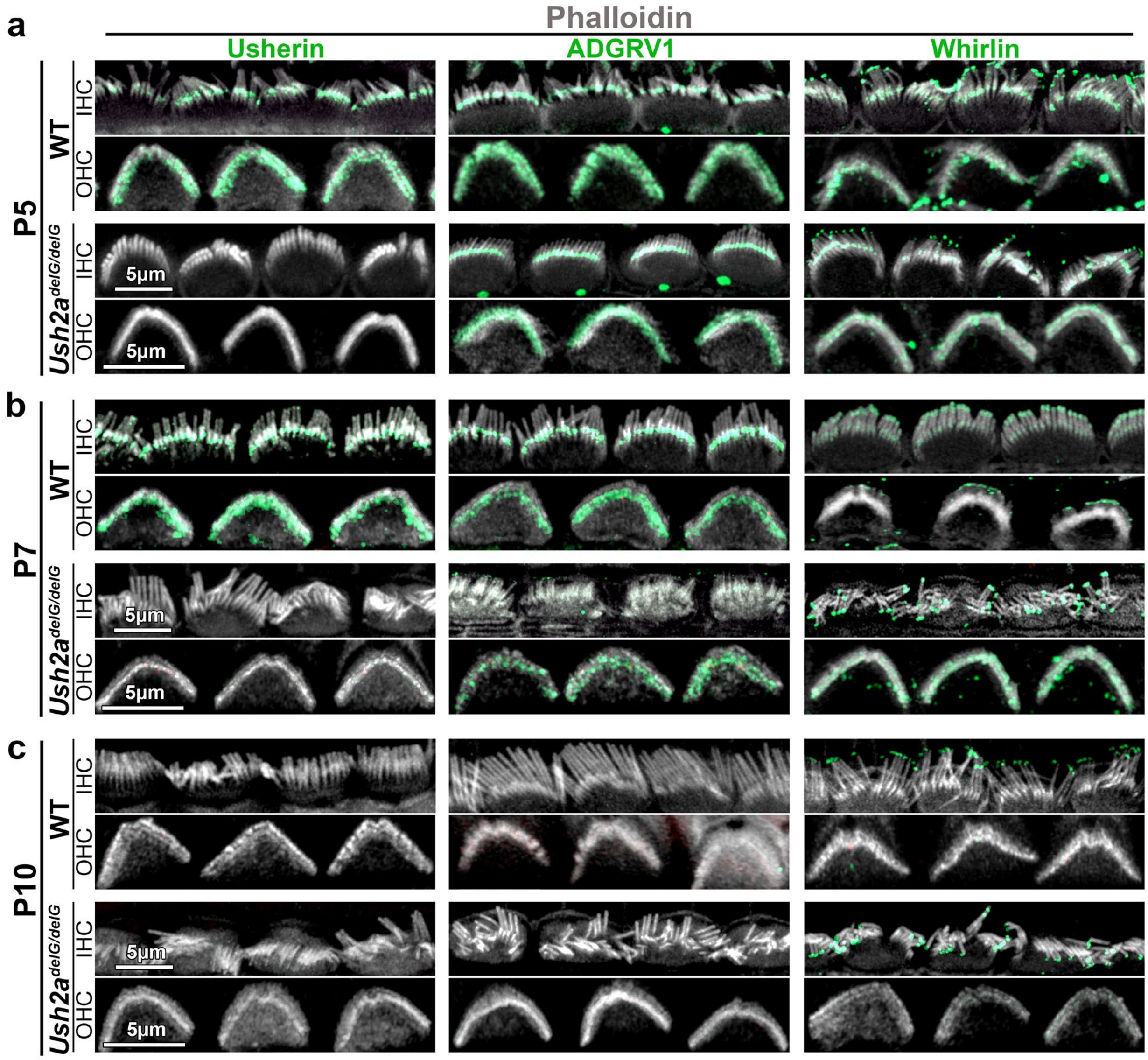

Fig. S4. Localization of Ush2 proteins throughout the development of IHC and OHCs in WT and *Ush2a*$^{delG/delG}$. Whole mount cochlea staining with phalloidin (grey), usherin (green), ADGRV1 (green), and whirlin (green) at P5 (**a**), P7 (**b**) and P10 (**c**). Both IHCs and OHCs for WT (upper images) and *Ush2a*$^{delG/delG}$ (lower images) are shown.

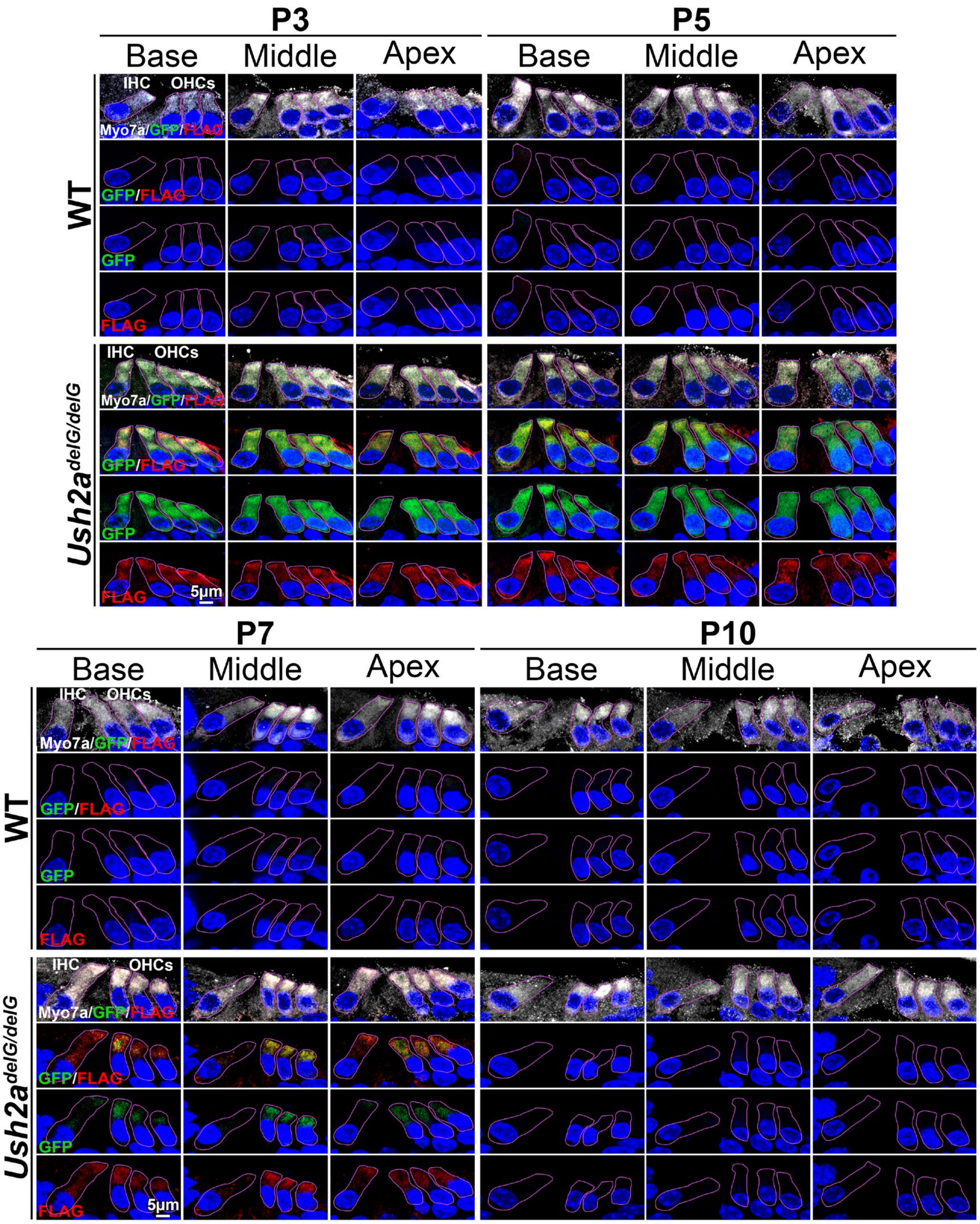

Fig. S5. Localization of mutant usherin using anti FLAG antibody and anti GFP antibody during development in WT and *Ush2a*$^{delG/delG}$ through different regions. Cochlea cryo-section stained with anti-myosin7a (grey and hair cells outlined in pink) and anti-FLAG (red) found within the cell bodies until P7 and GFP (green) found within the cell bodies until P7 of the hair cells in *Ush2a*$^{delG/delG}$.

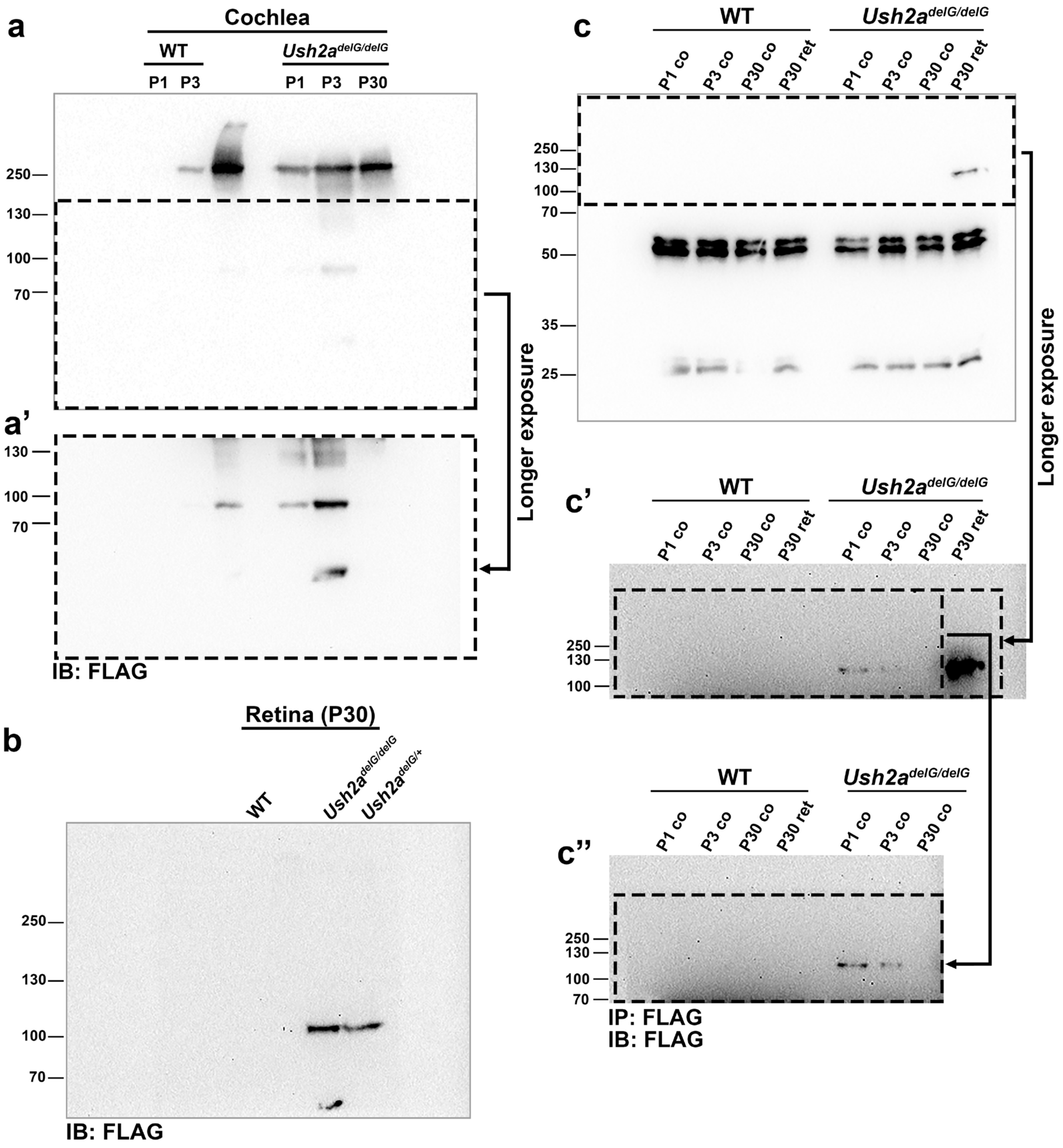

Fig. S6. Uncropped blots used to assemble Fig. 1c (a, a’ and b) and Fig. 1d (c, c’ and c’’). (**a**) Uncropped blot showing a non-specific band at 250 kDa. To visualize the 100 kDa band, the marked portion of the blot was exposed longer and presented in **a’**. (**b**) Uncropped blot showing the expression of mutant protein in the retina and used to build Fig. 1c. (**c**) Uncropped blot from the immunoprecipitation experiment shown in Fig. 1d. Shown are the bound from P1, P3 and P30 cochlea (co) and P30 retinal (ret) extracts from WT and *Ush2a*$^{delG/delG}$. Since the same antibody was used in IP and IB, strong antibody bands were seen at 50 and 25 kDa. To visualize the band

specific for the mutant protein, the antibody bands were masked and the marked portion of the blot was exposed for longer. (**c'**) Longer exposed image of the marked area in **c**. (**c''**) The retinal band from *Ush2a*$^{delG/delG}$.was masked from blot in c' to further exposed the marked area to show the bands in tP1 and P3 *Ush2a*$^{delG/delG}$ cochlea.

Supplementary References:

1 Zou, J. *et al.* Individual USH2 proteins make distinct contributions to the ankle link complex during development of the mouse cochlear stereociliary bundle. *Hum Mol Genet* **24**, 6944-6957, doi:10.1093/hmg/ddv398 (2015).

2 Reiners, J. *et al.* Scaffold protein harmonin (USH1C) provides molecular links between Usher syndrome type 1 and type 2. *Hum Mol Genet* **14**, 3933-3943, doi:10.1093/hmg/ddi417 (2005).
