# Supplementary material for: Expression of the human usherin c.2299delG mutation leads to early-onset auditory loss and stereocilia disorganization
Source: Commun Biol. 2023 Sep 12;6:933. doi: 10.1038/s42003-023-05296-x (PMC10497539; doi:10.1038/s42003-023-05296-x)
Supplement: Supplementary file 2 — Description of Additional Supplementary Files [file 42003_2023_5296_MOESM2_ESM.pdf]

## Description of Additional Supplementary Files

**File name:** Supplementary Data 1
**Description:** The source data for all graphs.
