# Supplementary material for: Expression of the human usherin c.2299delG mutation leads to early-onset auditory loss and stereocilia disorganization
Source: Commun Biol. 2023 Sep 12;6:933. doi: 10.1038/s42003-023-05296-x (PMC10497539; doi:10.1038/s42003-023-05296-x)
Supplement: Supplementary file 4 — Reporting Summary [file 42003_2023_5296_MOESM4_ESM.pdf]

nature portfolio

Corresponding author(s): Muna Naash

Last updated by author(s): Aug 24, 2023

Reporting Summary

Nature Portfolio wishes to improve the reproducibility of the work that we publish. This form provides structure for consistency and transparency in reporting. For further information on Nature Portfolio policies, see our Editorial Policies and the Editorial Policy Checklist.

Statistics

For all statistical analyses, confirm that the following items are present in the figure legend, table legend, main text, or Methods section.

n/a	Confirmed	
☐	☒	The exact sample size (*n*) for each experimental group/condition, given as a discrete number and unit of measurement
☐	☒	A statement on whether measurements were taken from distinct samples or whether the same sample was measured repeatedly
☐	☒	The statistical test(s) used AND whether they are one- or two-sided<br>*Only common tests should be described solely by name; describe more complex techniques in the Methods section.*
☐	☒	A description of all covariates tested
☐	☒	A description of any assumptions or corrections, such as tests of normality and adjustment for multiple comparisons
☐	☒	A full description of the statistical parameters including central tendency (e.g. means) or other basic estimates (e.g. regression coefficient) AND variation (e.g. standard deviation) or associated estimates of uncertainty (e.g. confidence intervals)
☐	☒	For null hypothesis testing, the test statistic (e.g. *F*, *t*, *r*) with confidence intervals, effect sizes, degrees of freedom and *P* value noted<br>*Give P values as exact values whenever suitable.*
☒	☐	For Bayesian analysis, information on the choice of priors and Markov chain Monte Carlo settings
☒	☐	For hierarchical and complex designs, identification of the appropriate level for tests and full reporting of outcomes
☒	☐	Estimates of effect sizes (e.g. Cohen's *d*, Pearson's *r*), indicating how they were calculated

Our web collection on statistics for biologists contains articles on many of the points above.

Software and code

Policy information about availability of computer code

Data collection	ABR: Tucker-Davis Technologies<br>DPOAE: Tucker-Davis Technologies<br>Microscopy, IHC: Zen 2.3, Version 2.3.64.0, Zeiss GmbH<br>Immuno blots: ImageLab 6.1, Version 6.1.0 build 7, BioRad Laboratories<br>qRT-PCR: CFX Manager, Version 2.1.1022.0523, BioRad Laboratories
Data analysis	ABR & DPOAE: Tucker-Davis Technologies<br>ABR & DPOAE sorting of dataset: Excel 2016, Version 16.0.5378.1000, Microscoft<br>Immuno blots: ImageLab 6.1, Version 6.1.0 build 7, BioRad Laboratories<br>qRT-PCR: CFX Manager, Version 2.1.1022.0523, BioRad Laboratories<br>qRT-PCR, sorting of dataset: Excel 2016, Version 16.0.5378.1000, Microscoft<br>Graphs and statistics: Prism 7, Version 7.05, GraphPad Software, Inc.<br>Assembling of Schematic: BioRender, biorender.com<br>Assembling of figures: Photoshop 2020, Version 21.0.3<br>IHC length measurements & OHC cell count: ImageJ, Version 1.53f51, NIH

For manuscripts utilizing custom algorithms or software that are central to the research but not yet described in published literature, software must be made available to editors and reviewers. We strongly encourage code deposition in a community repository (e.g. GitHub). See the Nature Portfolio guidelines for submitting code & software for further information.

Data

Policy information about availability of data

All manuscripts must include a data availability statement. This statement should provide the following information, where applicable:

- Accession codes, unique identifiers, or web links for publicly available datasets
- A description of any restrictions on data availability
- For clinical datasets or third party data, please ensure that the statement adheres to our policy

All data generated or analyzed during this study are included in this article and in its supplementary information file. The source data for all graphs is provided as a compiled Excel sheet (filename: Supplementary Data 1). Uncropped blots for Fig. 1c and Fig. 1d are shown in Fig. S6.

Research involving human participants, their data, or biological material

Policy information about studies with human participants or human data. See also policy information about sex, gender (identity/presentation), and sexual orientation and race, ethnicity and racism.

Reporting on sex and gender	N/A
Reporting on race, ethnicity, or other socially relevant groupings	N/A
Population characteristics	N/A
Recruitment	N/A
Ethics oversight	N/A

Note that full information on the approval of the study protocol must also be provided in the manuscript.

Field-specific reporting

Please select the one below that is the best fit for your research. If you are not sure, read the appropriate sections before making your selection.

☒ Life sciences ☐ Behavioural & social sciences ☐ Ecological, evolutionary & environmental sciences

For a reference copy of the document with all sections, see nature.com/documents/nr-reporting-summary-flat.pdf

Life sciences study design

All studies must disclose on these points even when the disclosure is negative.

Sample size	The sample size of each individual experiment are stated in the manuscript. For qualitative experiments a sample size of at least three (repeated on at least three separate occasions) was chosen based on our prior work. In qualitative experiments, the changes we describe are visually evident (e.g. a band shift on a western blot in deglycosylation experiments) and were repeatable through different replicates. For quantitative experiments, we did not perform sample size/power analysis calculations prior to the start of the study. Sample sizes are typically larger for physiological and functional measurements (such as ABD and DPOAE) where past experience has shown there is more inter-animal variability. In past functional studies, sample sizes in the range of 5-10 animals per group have been sufficient to detect (with statistical significance) differences between means on the order of ~15-20%, so we use that as a starting target goal for each sample size. In practice, many sample groups for physiological studies are larger, this arises due to random differences in group size (when a cohort of animals reaches the right age we typically perform analyses on all animals available at that time point to avoid potential bias from selecting only certain animals from the cage/cohort for evaluation). In this study, since it was difficult to predict the differences between group means and the within-group variance, we erred toward slightly larger sample sizes. For structural studies (e.g. IHC organization and OHC cell count) on inbred genetically modified mouse models, we have historically observed much less between-animal variation than with functional studies, and sample sizes of 3-5 have been sufficient to detect a difference (e.g. increased disorganization of IHCs), so that was what we used for this study.
Data exclusions	Minor exclusions in functional testing were done based on reasonable assumptions by comparing the auditory response between one ear to the other of the same mouse. If the waveforms generated by one ear significantly differed from the other (e.g. looked severely altered or lower waveform amplitude), then some exclusions of the animal were considered provided some issue with the ear itself was observed as well (e.g. ripped or torn ear)
Replication	At least three replicates were made. For quantitative experiments a bigger sample size was selected when possible. Sample sizes are stated in submitted study for each experiment. The observation stated in the study were reproducible.
Randomization	This study does not include treatments, so there is no randomization of animals into treatment vs. control groups. Instead the comparisons are between WT and Ush2adelG/delG animals, and as a result assignment to the control (WT) or experimental (Ush2adelG/delG) was made based on genotype. Age was another experimental variable, so this was controlled for directly (i.e. age-matched experiments between WT

and Ush2adelG/delG animals). Animals were raised under the same lighting, housing, and feeding conditions, and were on the same genetic background. Both sexes of animals were used in each experiment and we did not observe any difference in the distribution of males/female in each genotype.

Blinding

For all functional (i.e. ABR and DPOAE) and structural (i.e. histology, immunofluorescence, associated microscopy, and subsequent quantitative analysis) animals were only identified by tag number, not genotype or age. For ABR and DPOAE studies, all animals ready at a given time point were analyzed together and were unblinded to group/age only after data collection and analysis (e.g. measurement of amplitudes etc.) were completed. For structural studies such as histology, immunofluorescence, microscopy, and subsequent quantitative analysis (IHC disorganization and OHC cell count) where it was important that each experiment contain representatives of each group, selected tissue sections based on the group (i.e. to ensure that all groups were in the experiment), and then identified only by tag number for the labeling/imaging/analysis. Only after all this was completed were the tag numbers unblinded for statistical analysis and assembly of figures. The only time unblinding (to group) was done prior to the final analysis was for western blots where samples were extracted and protein concentrations measured while identified only by tag number but groups were unblinded prior to loading on the gel so samples could be loaded in a logical order.

Reporting for specific materials, systems and methods

We require information from authors about some types of materials, experimental systems and methods used in many studies. Here, indicate whether each material, system or method listed is relevant to your study. If you are not sure if a list item applies to your research, read the appropriate section before selecting a response.

Materials & experimental systems

n/a	Involved in the study	
☐	☒	Antibodies
☒	☐	Eukaryotic cell lines
☒	☐	Palaeontology and archaeology
☐	☒	Animals and other organisms
☒	☐	Clinical data
☒	☐	Dual use research of concern
☒	☐	Plants

Methods

n/a	Involved in the study	
☒	☐	ChIP-seq
☒	☐	Flow cytometry
☒	☐	MRI-based neuroimaging

Antibodies

Antibodies used

Secondary Antibodies:
Alexa Fluor-555, donkey-anti-rabbit, Catalog number: A32794, Lot number: TI271031, Supplier: Invitrogen
Alexa Fluor-594, donkey-anti-rat, Catalog number: A21209, Lot number: 1547508, Supplier: Life Technologies
Goat Anti-Rabbit IgG Antibody, HRP conjugate, Catalog number: AP187P, Lot number: 2709676, Supplier: Sigma-Aldrich
Primary Antibodies:
Provided in supplementary table 1

Validation

VLGR1 (rabbit): validated in: Reiners, J. et al. Scaffold protein harmonin (USH1C) provides molecular links between Usher syndrome type 1 and type 2. Hum Mol Genet 14, 3933-3943, (2005).

Proteintech, Rabbit primary antibodies (WHRN (25881-1-AP) used in IF)
Validation for IF: The target protein is knocked out via siRNA in tissue culture. ICC is performed to check for absence of labeling in the knockout cells.

Usherin b: validated in: Zou, J. et al. Individual USH2 proteins make distinct contributions to the ankle link complex during development of the mouse cochlear stereociliary bundle. Hum Mol Genet 24, 6944-6957, (2015).

Biolegend, Rat primary antibody (FLAG (637301 used in IF)
Validation for IF: The target protein is either knocked down via siRNA or knocked out via CRISPR/Cas9 in tissue culture. ICC of these cells is checked for reduced or absent labeling in the knockdown and knockout scenario, respectively, when compared to the WT. This can be combined with exogenous expression of the target protein in cell lines which do not express the target protein endogenously. Transfection of exogenous protein is followed by knockdown/knockout.

Abcam, Rabbit primary antibodies (Myosin VIIa/MYO7A antibody (ab3481) used only in IF)
Validation for IF: The target protein is either knocked down via siRNA or knocked out via CRISPR/Cas9 in tissue culture. ICC of these cells is checked for reduced or absent labeling in the knockdown and knockout scenario, respectively, when compared to the WT.

Animals and other research organisms

Policy information about studies involving animals; ARRIVE guidelines recommended for reporting animal research, and Sex and Gender in Research

Laboratory animals

Species: Mus Musculus; Strain: C57BL/6J; Age: P1-P500, specific ages for each experiment are stated in submitted manuscript. Mice were kept at an ambient temperature of 22 C and a humidity of 50%.

Wild animals	No wild animals were used in this study.
Reporting on sex	Initial experiments showed no sex specific cochlear phenotype. Thus, for the experiments described in the submitted sex was not considered and male and female animals were used. No data was collected counting the amount of female vs. male animals used for each experiment. Instead only the total number of used animals is stated.
Field-collected samples	No field-collected samples were used in this study.
Ethics oversight	All handling, maintenance, and experimental use of animals followed protocols approved by the University of Houston's Institutional Animal Care and Use Committees and were performed according to the NIH and the Association for Research in Vision and Ophthalmology (ARVO) guidelines. Protocol: PROTO201800045.

Note that full information on the approval of the study protocol must also be provided in the manuscript.
